# Simultaneous Determination of Methylated Nucleosides by HILIC–MS/MS Revealed Their Alterations in Urine from Breast Cancer Patients

**DOI:** 10.3390/metabo12100973

**Published:** 2022-10-14

**Authors:** Zhihao Fang, Yiqiu Hu, Xiujuan Hong, Xiaoxiao Zhang, Tao Pan, Chi Pan, Shu Zheng, Cheng Guo

**Affiliations:** 1 Cancer Institute (Key Laboratory of Cancer Prevention and Intervention, China National Ministry of Education), The Second Affiliated Hospital, Zhejiang University School of Medicine, Hangzhou 310009, China; 2 Department of Breast Surgery, The Second Affiliated Hospital, Zhejiang University School of Medicine, Hangzhou 310009, China; 3 Cancer Center, Zhejiang University, Hangzhou 310058, China

**Keywords:** HILIC–MS/MS, methylated nucleosides, breast cancer, biomarker, urine

## Abstract

RNA methylation plays a vital role in the pathogenesis of a variety of diseases including cancer, and aberrant levels of modified nucleosides in RNA were revealed to be related to cancer. Urine is a favored source for biomarker discovery due to the non-invasion to patients. Herein, we developed a sensitive hydrophilic interaction liquid chromatography tandem mass spectrometry (HILIC–MS/MS) method combined with stable isotope dilution for accurate quantification of methylated nucleosides in human urine. With this method, we successfully quantified ten methylated nucleosides in urine samples collected from healthy controls and breast cancer patients. We found *N*^6^-methyladenosine (m^6^A), 2′-O-methyladenosine (A_m_), *N*^1^-methyladenosine (m^1^A), *N*^6^,2′-O-dimethyladenosine (m^6^A_m_), *N*^1^-methylguanosine (m^1^G), 2′-O-methylguanosine (G_m_), 5-methylcytidine (m^5^C) and 2′-O-methylcytidine (C_m_) were all decreased in early-stage breast cancer patients, and a nomogram prediction model was constructed. Locally advanced breast cancer patients exhibited elevated levels of urinary 2′-O-methylated nucleosides in comparison to early-stage breast cancer patients. Together, we developed a robust method for the simultaneous determination of methylated nucleosides in human urine, and the results revealed an association between the contents of urinary methylated nucleosides and the occurrence of breast cancer, which may stimulate future studies about the regulatory roles of these methylated nucleosides in the initiation and progression of breast cancer.

## 1. Introduction

Breast cancer is the most prevalent cancer around the world, despite great advances in treatment, and has become the leading cause of cancer-associated death among women [1]. The high mortality of breast cancer is mainly due to late detection and untimely prediction of its progression. Due to the inconvenience of ultrasonography/mammography, the low specificity of serum breast cancer biomarker (i.e., CA-153) and the invasiveness of biopsy, a number of breast cancer patients refuse to be examined, which leads to delay in diagnosis. Early-stage breast cancer (EBC) patients are potentially exempt from chemotherapy, while once developed to locally advanced breast cancer (LABC), surgery combined with chemotherapy is required. Chemotherapy regimens for breast cancer often contain paclitaxel or docetaxel, which can cause severe hair loss and even permanent chemotherapy-induced alopecia [2]. Therefore, it is necessary to hunt for convenient, non-invasive and highly specific biomarkers for the early detection and progression of breast cancer.

Blood and urine are two common sources of body fluids for biomarker discovery. Urine, compared to blood, is clinically more accessible and noninvasive. Urine excretion of methylated nucleosides can reflect systemic degradation and/or turnover of methylated RNA, especially tRNA. RNA methylation plays important roles in multiple biological activities and abnormal methylation levels are associated with cancers. Urinary methylated nucleosides were proposed to have potential as biomarkers in different types of cancers such as colorectal cancer [3,4], gastric cancer [4,5,6], renal cancer [7], bladder cancer [8], ovarian cancer [9] and breast cancer [10,11,12,13,14,15]. In recent years, several studies found that the levels of *N*^1^-methyladenosine (m^1^A) and *N*^1^-methylguanosine (m^1^G) were elevated in urine from breast cancer patients [11,12,13]. Additionally, the level of *N*^1^-methylinosine (m^1^I) in urine was also found to be higher in breast cancer patients [10,13,14]. Zheng et al. found that the levels of urinary *N*^6^-methyladenosine (m^6^A), 5-methylcytidine (m^5^C) and *N*^2^-methylguanosine (m^2^G) were increased in breast cancer patients [10]. Another study found that elevated 3-methylcytidine (m^3^C) levels in the urine could be used to diagnose breast cancer [15]. There are many kinds of methylated nucleosides, and exploration of the relationship between the urinary methylated nucleosides and the early detection and progression of breast cancer is desirable.

Since urine contains endogenous interferences including proteins, metabolites and inorganic salts, the determination of the urinary nucleosides usually requires urine sample pretreatment by solid-phase extraction (SPE) using various sorbents such as octadecylsilane (C18) [16], phenylboronic acid (PBA) [17,18], hydrophilic-lipophilic balance (HLB) [19,20] and so on. However, SPE methods are costly and time-consuming, and the sorbents have the limitation of poor selectivity. For instance, PBA cartridges cannot retain analytes without *cis*-diol groups (e.g., 2′-O-methylated nucleosides [21]). In addition, HLB and C18 cartridges exhibited poor adsorption of cytosine [3] and guanosine [22] modifications, respectively. Liquid chromatography tandem mass spectrometry (LC–MS/MS) plays a crucial role in the realm of biomarker discovery [23,24,25,26,27,28]. As a complementary tool of reversed-phase liquid chromatography tandem mass spectrometry (RPLC–MS/MS), hydrophilic interaction liquid chromatography tandem mass spectrometry (HILIC–MS/MS), which mainly uses organic solvents as mobile phases, has higher sensitivity and has been widely utilized in biological sample analysis in recent years [29,30].

Herein, we established a rapid, convenient and cost-effective liquid–liquid extraction procedure for the extraction of methylated nucleosides from human urine samples. We also developed a sensitive and accurate HILIC–MS/MS method by using malic acid as a mobile phase additive and utilizing a stable isotope dilution strategy for the simultaneous determination of ten methylated nucleosides in urine samples from healthy volunteers and breast cancer patients. 

## 2. Materials and Methods

### 2.1. Chemicals and Reagents

Chromatographic grade acetonitrile was bought from Merck KGaA (Darmstadt, Germany). Formic acid was obtained from Fluka (Muskegon, MI, USA) and water was obtained from a Milli-Q water purification apparatus (Millipore, Milford, MA, USA). Malic acid, ammonium formate and m^5^C were bought from Sigma-Aldrich (St Louis, MO, USA). Nine other methylated nucleosides, [D_3_]m^6^A, [D_3_]m^1^A, [D_3_]m^6^A_m_, [D_3_]U_m_, [^13^C_5_]m^5^U, [^13^C^15^N_2_]G and [^13^C_5_]C, were purchased from Toronto Research Chemicals (Toronto, Canada); [^13^C_5_]A_m_ and [^13^C_5_]m^5^C were synthesized previously [3,4].

### 2.2. Standard Preparation

Ten nucleoside standards and isotope-labeled internal standards (IS) were stored at a concentration of 5 mM or 1 mM. Mixed working standard solutions of these ten methylated nucleosides (10 μM for m^1^A, m^1^G and C_m_, and 1 μM for other nucleosides) were obtained by diluting the preserved standard solution with water. The mixed standard solution was gradually diluted (1, 2.5, 5, 10, 25, 50, 100, 250, 500, 1000, 2500, 5000 nM) and spiked with IS mixed solution (200 nM of [D_3_]m^6^A, 5 nM of [^13^C_5_]A_m_, 1000 nM of [D_3_]m^1^A, 20 nM of [D_3_]m^6^A_m_, 100 nM of [^13^C^15^N_2_]m^1^G, 100 nM of [^13^C^15^N_2_]G_m_, 1 nM of [^13^C_5_]m^5^C, 100 nM of [^13^C_5_]C_m_, 20 nM of [^13^C_5_]m^5^U and 200 nM of [D_3_]U_m_) for the establishment of calibration curves. Quality control (QC) samples at low, medium and high concentrations were prepared for the evaluation of stability, precision and accuracy. 

### 2.3. Sample Collection

This study was carried out with the approval of the Ethics Committee of Medical Research of the Second Affiliated Hospital, Zhejiang University School of Medicine (SAHZU). A total of 109 healthy controls (mean age of 47.0 ± 8.6 years, range from 35–69 years), 128 patients with EBC (mean age of 53.0 ± 11.8 years, range from 36–75 years) and 91 patients with LABC (mean age of 53.2 ± 10.7 years, range from 38–75 years) were recruited from SAHZU. EBC includes carcinoma in situ and stage I, and LABC means stage II and stage III. The staging is based on the 8th edition of AJCC guidelines. All breast cancer patients were confirmed by a pathological diagnosis, did not receive any cancer-related treatment and did not have other types of cancer or any other metabolic diseases. All participants signed informed consent materials in advance. The midstream urine samples were collected and stored at −80 °C. We calibrated the nucleoside levels by measuring urinary creatinine with the help of the Department of Laboratory Medicine, SAHZU.

### 2.4. Sample Pretreatment

At first, the urine sample was naturally thawed and centrifuged at 13,000 rpm at 4 °C for 15 min. Next, 10 μL of supernatant was spiked with 10 μL of IS solution and 180 μL of acetonitrile and placed in −20 °C for 1 h after 10 s of shock. After centrifugation under the same conditions for 5 min, 180 μL of supernatant was aspirated and dried under vacuum. Then, 90 μL of acetonitrile:H_2_O 9:1 (*v/v*) solution was used to redissolve the dried samples. After shaking for 10 s, sonication for 15 s and centrifugation for 5 min, 80 μL of the supernatant were aspirated into the sample vials for HILIC–MS/MS detection.

### 2.5. HILIC–MS/MS Analysis

An Acquity UPLC system (Waters, Milford, MA, USA) and a BEH Amide column (100 mm × 2.1 mm, 1.7 μm) were used for chromatographic separation. Two solutions were prepared and used as mobile phases. Eluent A was H_2_O containing 0.2% formic acid, 10 mM ammonium formate and 0.06 mM malic acid; eluent B was acetonitrile containing 0.2% formic acid, 2 mM ammonium formate and 0.06 mM malic acid. We set the flow rate at 0.4 mL/min and the LC gradient program was as follows: 0–4.0 min, 6% A; 4.0–6.0 min, 6–25% A; 6.0–6.5 min, 25–6% A; 6.5–8.0 min, 6% A. The samples were stored at 4 °C and the column was set at 40 °C. The injection volume was 5 μL. A switching valve was used to reduce interference by introducing the eluent from the column to the ion source during 1.0–7.0 min. Each sample was measured twice to take the average.

A 4000 QTRAP mass spectrometer (AB SCIEX, Foster City, CA, USA) was applied for MS detection. Multiple reaction monitoring (MRM) mode was used to quantify these modified nucleosides by monitoring the corresponding ion transitions. The ion source temperature and spray voltage were set at 550 °C and 5.5 kV, respectively. The ion source gases 1 and 2 and curtain gas were set at 45, 45 and 50 psi, respectively. The MRM parameters were optimized by directly injecting standard solutions into the mass spectrometer using a peristaltic pump. The ion transitions of these ten nucleosides and their corresponding IS, along with the optimized MRM parameters, were listed in Table S1.

### 2.6. Method Validation

The calibration curves were plotted by comparing the peak area ratio and concentration ratio of the analyte/IS. They can be represented by the formula as y = ax + b, where x is the concentration ratio of analyte/IS and y is the peak area ratio of analyte/IS. The limit of detection (LOD) and limit of quantification (LOQ) of each analyte were defined as the concentration with a signal-to-noise ratio of 3 and 10, respectively. The quality control (QC) samples were prepared in triplicate at three different levels and were measured within 1 day and in 3 consecutive days, respectively. The accuracy referred to the ratio of measured/theoretical concentration. The recovery (R) of extraction was evaluated by adding three different levels of standard solutions into urine samples and recovery was determined by comparing the measured concentration difference to the actually added concentration. The stability was examined by detecting the concentrations of QC samples that have been placed for a period of time at different temperatures versus the concentrations of fresh samples. The matrix effect was obtained by comparing the slope ratios of the calibration curves constructed in urine extracts and pure solvent.

### 2.7. Statistical Analysis

Statistical analysis of data was performed using IBM SPSS Statistics 24.0 software (IBM, Armonk, NY, USA) and R version 4.1.0 software (The R Foundation for Statistical Computing, Austria, Vienna). The differences of analyte concentration between normal controls and breast cancer patients were accessed by Mann–Whitney *U* test and Chi-square test, where *p* value less than 0.05 was considered meaningful. Forward stepwise logistic regression analyses were applied for identifying predictive factors of early breast cancer diagnosis, and a nomogram was then established based on the logistic model.

## 3. Results and Discussion

### 3.1. Synthesis and Characterization of Stable Isotope-Labeled G_m_, m^1^G and C_m_

**Figure 1:**
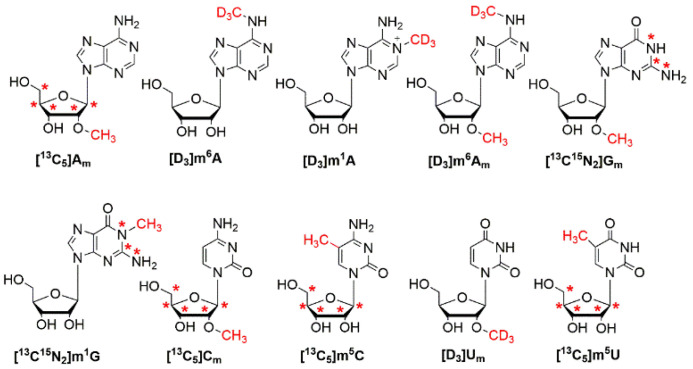
The chemical structures of stable isotope-labeled A_m_, m^6^A, m^1^A, m^6^A_m_, G_m_, m^1^G, C_m_, m^5^C, U_m_ and m^5^U. Asterisk (*) indicates the site of ^13^C or ^15^N labeling.

In this study, we aimed to unambiguously identify and accurately quantify the methylated nucleoside modifications, including A_m_, m^6^A, m^1^A, m^6^A_m_, G_m_, m^1^G, C_m_, m^5^C, U_m_ and m^5^U in human urine by utilizing a stable isotope dilution strategy. Since there are no commercially available stable isotope-labeled G_m_, m^1^G and C_m_, we first synthesized the isotope-labeled G_m_, m^1^G and C_m_ according to the established method with minor modifications [31]. The structures of these ten stable isotope-labeled nucleosides are shown in Figure 1. We purified [^13^C^15^N_2_]m^1^G, [^13^C^15^N_2_]G_m_ and [^13^C_5_]C_m_ by HPLC and they were characterized by high-resolution mass spectrometry (Figure S1). The detailed procedures for the syntheses and purifications are described in the Supplementary Materials (Method S1).

### 3.2. Establishment of HILIC–MS/MS Method for the Simultaneous Determination of A_m_, m^6^A, m^1^A, m^6^A_m_, G_m_, m^1^G, C_m_, m^5^C, U_m_ and m^5^U

**Figure 2:**
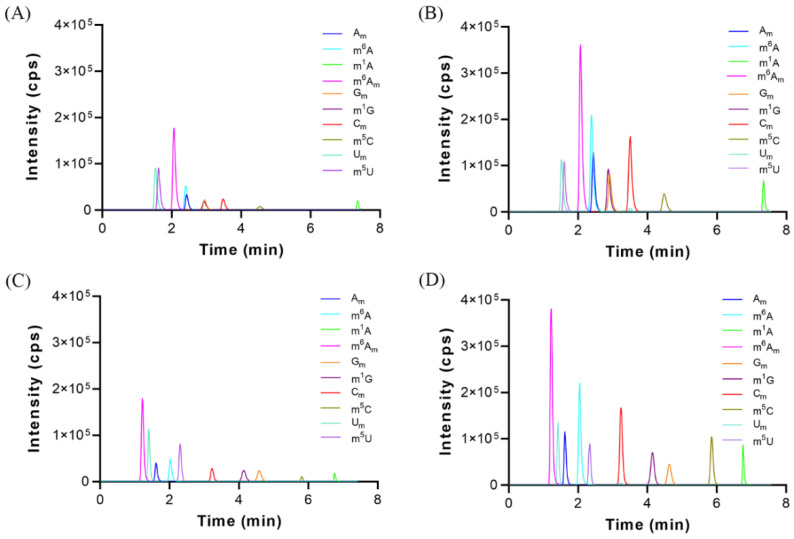
The multiple reaction monitoring (MRM) chromatograms under different conditions. (**A**) BEH HILIC column, without the use of malic acid, (**B**) BEH HILIC column, with the use of malic acid, (**C**) BEH Amide column, without the use of malic acid, and (**D**) BEH Amide column, with the use of malic acid. The concentrations of U_m_ and m^5^U were 1000 nM, and the concentrations of other nucleosides standards were 20 nM. The injection volume was 5.0 μL.

Our recent study demonstrated that the sensitivity of determination of modified cytosine nucleosides could be dramatically improved by using malic acid as a mobile phase additive in HILIC–MS/MS analysis [3]. Thus, we tested whether malic acid could improve the HILIC–MS/MS detection of these methylated nucleosides. As shown in Figure 2A,B, the detection of these methylated nucleosides was significantly improved with the utilization of 0.06 mM malic acid. However, these nucleoside modifications, especially for purine nucleoside modifications, could not be completely separated even under optimized chromatographic conditions when a bare silica phase BEH HILIC column (2.1 × 100 mm, 1.7 μm) was used. Hence, another type of HILIC column, amide bonded column (i.e., BEH Amide column, 2.1 × 100 mm, 1.7 μm) was tested. As illustrated in Figure 2C,D, satisfactory separation of these methylated nucleosides was achieved, and thus BEH Amide column was selected for analysis. We found that the MRM signals were dramatically elevated when malic acid was used, and the LOD values of most of these nucleosides could reach the sub-femtomole level (Table S2), which were lower than those previously reported [32,33,34] and indicated that excellent sensitivity was obtained. In addition, the developed analytical method is very rapid and the measurement could be finished within 7 min, which implies that this method is suitable for large-scale clinical samples analysis.

### 3.3. Urine Samples Pretreatment

Urine samples contain many inorganic salts that may suppress the ionization of analytes in MS. Hence, desalting urine samples is required before LC–MS/MS detection. As a powerful pretreatment technique, SPE was commonly used for desalting urine samples. Nevertheless, it has some drawbacks, e.g., high cost of cartridges, poor selectivity and a tedious process. Herein, a simple liquid–liquid extraction step was utilized. Acetonitrile was used as the extraction solvent and the volume ratio of sample to solvent was set at 1:9 (*v/v*). After centrifugation at 13,000 rpm at 4 °C for 5 min, the mixture was divided into two layers and the upper organic layer was aspirated and evaporated. Meanwhile, the proteins in urine samples could also be precipitated and removed by acetonitrile. Hence, a quick, convenient and cost-effective sample preparation step could be applied for the pretreatment of more than 300 urine samples in this study. 

### 3.4. Validation of the Analytical Method

In the light of the aforementioned procedures, ten calibration curves were constructed. Each of them exhibited excellent linearities (R^2^ > 0.999) in different analytical ranges, and the results are illustrated in Table 1. For matrix effect, the slope ratio values observed were in the range of 94.4–107.7% (Table 1), which demonstrated the interference of matrix on the determination of these methylated nucleosides was negligible. As shown in Table S3, the accuracy of the intra- and inter-day analysis ranged from 90.18-109.48%. The intra-day precision value was within 9.6% and the inter-day precision value was within 7.6%. These results indicate that sufficient reproducibility and accuracy were obtained. The recovery was evaluated and the values ranged from 98.04–114.01% (Table S4), which indicated that the liquid–liquid extraction method is effective. The percentages of analytes remaining after 24 h, 48 h and 72 h under different temperatures are shown in Table S5, and the results demonstrated that sample stability could be guaranteed. In summary, the developed HILIC –MS/MS method could meet the requirements of quantitative analysis of these methylated nucleosides in human urine samples, and it was simple, quick, sensitive, accurate and robust.

### 3.5. Identification of Methylated Nucleoside Modifications in Human Urine

**Figure 3:**
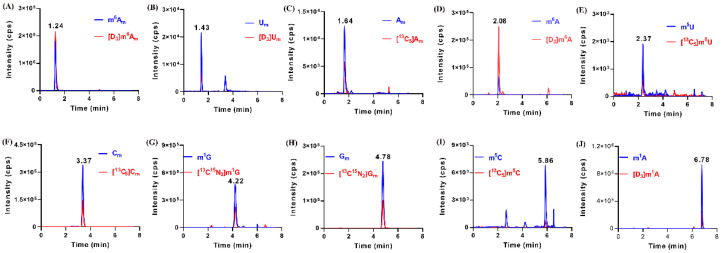
Representative multiple reaction monitoring (MRM) chromatograms of (**A**) m^6^A_m_, (**B**) U_m_, (**C**) A_m_, (**D**) m^6^A, (**E**) m^5^U, (**F**) C_m_, (**G**) m^1^G, (**H**) G_m_, (**I**) m^5^C, and (**J**) m^1^A and spiked isotope-labeled internal standards in a urine sample.

By using the established HILIC–MS/MS method, we detected these methylated nucleosides in urine samples from 109 healthy volunteers and 219 patients with breast cancer. The results showed that these methylated nucleosides were detected in most of the urine samples. In few urine samples, m^1^A and m^1^G could not be detected as a result of their extremely low amount. As illustrated in Figure 3, the retention time of m^6^A_m_, U_m_, A_m_, m^6^A, m^5^U, C_m_, m^1^G, G_m_, m^5^C and m^1^A were 1.24, 1.43, 1.64, 2.07, 2.37, 3.37, 4.22, 4.78, 5.86 and 6.78 min, respectively. Of note, all of the methylated nucleosides had the same retention time with their corresponding IS in MRM chromatograms, which confirmed the presence of these modifications in urine.

### 3.6. Quantification of Methylated Nucleoside Modifications in Human Urine

The contents of these nucleoside modifications in urine samples were calculated in the light of the calibration curves and calibrated by the urinary creatinine concentration. The normalized results showed that the measured levels of A_m_, m^6^A, m^1^A, m^6^A_m_, G_m_, m^1^G, C_m_, m^5^C, U_m_ and m^5^U in human urine ranged from 0.09–13.33, 0.61–72.55, 116.26–4230.32, 1.65–21.17, 20.47–467.21, 24.01–2680.74, 25.47–1431.11, 0.22–13.62, 12.80–275.32 and 2.00–26.74 nmol/mmol creatinine, respectively. The measured average levels of urinary methylated nucleosides in the normal controls (NC), EBC and LABC patients are shown in Table S6, and the detailed concentrations of methylated nucleosides and creatinine in each participant are listed in Table S7.

### 3.7. A Risk Nomogram for Prediction of the Occurrence of Early-Stage Breast Cancer

**Figure 4:**
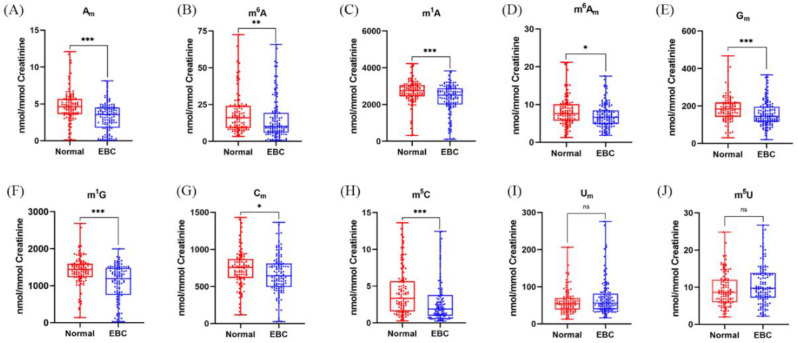
The calibrated concentrations of (**A**) A_m_, (**B**) m^6^A, (**C**) m^1^A, (**D**) m^6^A_m_, (**E**) G_m_, (**F**) m^1^G, (**G**) C_m_, (**H**) m^5^C, (**I**) U_m_, and (**J**) m^5^U in the urine samples of normal controls and EBC patients. (ns *p* > 0.05, * *p* < 0.05, ** *p* < 0.001, *** *p* < 0.0001).

Tumors in early-stage breast cancer are usually too small to be detected by ultrasound screening, and the discovery of novel biomarkers for detection of EBC is urgently needed. We compared the levels of these methylated nucleosides in EBC patients and healthy volunteers. As shown in Figure 4, it was obvious that the levels of A_m_, m^6^A, m^1^A, m^6^A_m_, G_m_, m^1^G, C_m_ and m^5^C in urine were decreased in patients with EBC in comparison to healthy controls, which indicated that lower levels of these nucleoside modifications in urine have a higher probability of breast cancer. However, the contents of U_m_ and m^5^U in urine had no significant differences between these two groups. 

Previous studies show that the levels of m^1^A, m^6^A and m^1^G in the urine of breast cancer patients were all higher than those in normal controls [10,11,12], which were inconsistent with our current results. This may be attributed to the utilization of different analytical methods. Moreover, we used stable isotope-labeled internal standards which could ensure accurate quantification. Recently, we revealed that the levels of A_m_, m^6^A, and m^6^A_m_ in urine from patients with breast cancer were higher than those in normal controls [35], which is different from the results shown in Figure 4. However, a limited number of samples were analyzed previously. In the present work, many more urine samples were analyzed and this led to more reliable statistic results. Besides, only early-stage breast cancer (stage 0 and stage 1) patients were compared with normal controls in this study, and this could also result in different conclusions from previous studies.

**Figure 5:**
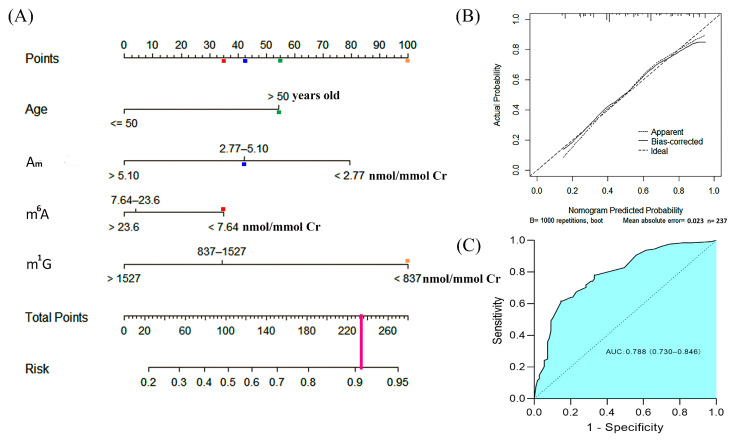
Construction of a predictive nomogram for the detection of early-stage breast cancer. ***(*A**) A nomogram for predicting the probability of the occurrence of early-stage breast cancer (EBC); (**B**) calibration plot. The reference line represents perfect equality of the predicted probability and the actual incidence of EBC, and (**C**) Receiver operating characteristics (ROC) curve of the multivariate logistic regression model. AUC: area under curve.

The levels of urinary methylated nucleosides were divided into three groups according to the quartiles, the cutoff values of A_m_, m^6^A, m^1^A, m^6^A_m_, G_m_, m^1^G, C_m_ and m^5^C are listed in Table S8. We performed univariate logistic analyses and found that individuals with age > 50 years old, body mass index > 24 kg/m^2^ or menarche age ≤ 12 years old had a higher possibility of occurrence of breast cancer, whereas CA-153 was not correlated with the occurrence of breast cancer (Table S9). Multivariate analyses confirmed age, A_m_, m^6^A and m^1^G were independent risk factors of breast cancer (Table S9). Therefore, age and these three urinary nucleosides were selected to construct a predictive nomogram for the early detection of breast cancer (Figure 5A). In this nomogram prediction model, each of these risk factors was assigned points. For instance, 55 points or zero point were assigned if the age was > or ≤50 years old, respectively; 80 points or zero point were assigned if the level of A_m_ was <2.77 or >5.10 nmol/mmol creatinine, respectively; finally, 42.5 points was assigned if the level of A_m_ was in the range from 2.77 to 5.10 nmol/mmol creatinine. The sum of these points corresponded to a probability of the occurrence of breast cancer. As an example, one patient was 56 years old and her urinary A_m_, m^6^A and m^1^G levels were 3.87, 6.64 and 657 nmol/mmol Cr, respectively, and thus 55, 42.5, 35 and 100 points were assigned, respectively (The colored boxes in Figure 5A). The total points were 232.5, which would give a predictive risk value of 0.91 for the occurrence of EBC. A calibration curve, based on internal validation with additional 1000 bootstraps, was constructed (Figure 5B). The outcomes of a Hosmer–Lemeshow test indicated a satisfactory fit between the prediction and the actual observation. In addition, the receiver operating characteristics (ROC) curve of the nomogram was shown in Figure 5C, and the area under curve (AUC) was 0.788 (95% CI, 0.730–0.846), which indicated a great discriminative ability.

### 3.8. Elevated Levels of 2′-O-Methylated Nucleoside Modifications in Urine Suggest the Progression of Early-Stage Breast Cancer

**Figure 6:**
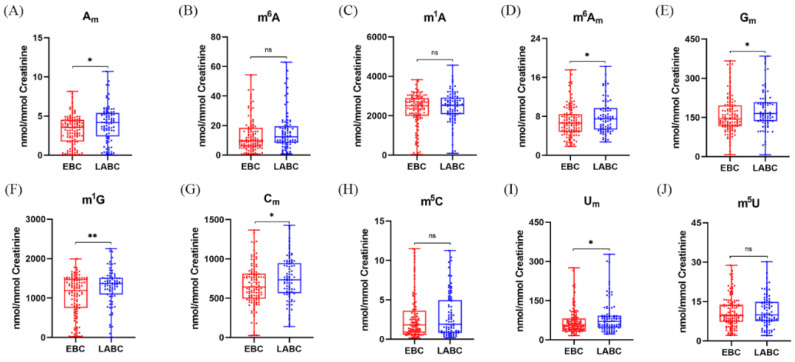
Quantification and statistical analysis results of (**A**) A_m_, (**B**) m^6^A, (**C**) m^1^A, (**D**) m^6^A_m_, (**E**) G_m_, (**F**) m^1^G, (**G**) C_m_, (**H**) m^5^C, (**I**) U_m_, and (**J**) m^5^U in urine from EBC and LABC patients. (ns *p* > 0.05, * *p* < 0.05, ** *p* < 0.001).

We revealed that the reduction of the levels of methylated nucleoside modifications in urine might be related to EBC, and we wondered whether the levels of methylated nucleoside modifications in urine would further decrease in LABC patients. Surprisingly, compared with patients with EBC, there was a significant increase in the levels of several methylated nucleoside modifications in urine from LABC patients. As demonstrated in Figure 6, the contents of A_m_, m^6^A_m_, G_m_, m^1^G, C_m_ and U_m_ in urine were obviously elevated in LABC patients. Notably, the majority of these modifications were 2′-O-methylation, which implies that an increase of urinary levels of 2′-O-methylated nucleosides in patients after the diagnosis of EBC may imply the progression of the cancer. From these, we speculate that RNA methylation has different mechanisms for the occurrence and progression of breast cancer, low levels of RNA methylation contribute to the occurrence of breast cancer, and elevated 2’-O methylation modifications are conducive to the progression of EBC. However, their inherent specific mechanisms need to be further explored.

### 3.9. Patients with Triple-Negative Breast Cancer Have Higher Levels of m^1^G and m^5^C in Urine

Triple-negative breast cancer (TNBC) has a significantly poorer prognosis than other breast cancer subtypes. A recent study showed that m^5^C and its regulators are related to the prognosis of TNBC [36]. To investigate whether there were differences in the levels of m^5^C and other methylated nucleosides in urine between TNBC and non-TNBC patients, we compared the levels of these nucleosides between these two groups. Our findings revealed that the contents of m^1^G and m^5^C in urine from TNBC patients were much higher than those in urine from non-TNBC patients (Figure S2). This indicates that the elevated m^1^G and m^5^C may be a distinct feature of TNBC, and it also implies the potential regulatory roles of these methylated modifications in the poor prognosis of TNBC.

## 4. Conclusions

In this work, a fast, sensitive and robust HILIC–MS/MS method was developed for the simultaneous quantification of ten methylated nucleosides in urine samples from 109 healthy controls and 219 breast cancer patients. Ten methylated nucleosides including A_m_, m^6^A, m^1^A, m^6^A_m_, G_m_, m^1^G, C_m_, m^5^C, U_m_ and m^5^U were identified and quantified. In addition, we constructed a nomogram for detection of early-stage breast cancer using age and three urinary methylated nucleosides (A_m_, m^6^A and m^1^G), and we also found the contents of 2′-O-methylted nucleoside modifications in urine were increased with the progression of breast cancer. Moreover, we revealed that the contents of m^1^G and m^5^C in urine were increased in TNBC patients in comparison to non-TNBC patients. All of these results indicate that these methylated nucleosides may serve as non-invasive biomarkers for the early detection and progression of breast cancer.

## Figures and Tables

**Table 1:** Linear equations and matrix effect of A_m_, m^6^A, m^1^A, m^6^A_m_, G_m_, m^1^G, C_m_, m^5^C, U_m_ and m^5^U in hydrophilic interaction liquid chromatography tandem mass spectrometry (HILIC–MS/MS) analysis.

	Linear Equation	R^2^ Value	Linear Range (nM)	Matrix Factor (%)
A_m_	y = 1.8251x + 0.0196	0.9993	1–50	109.22
m^6^A	y = 0.6465x + 0.0034	0.9998	1–250	101.78
m^1^A	y = 0.9332x + 0.0329	0.9996	1–10,000	94.42
m^6^A_m_	y = 1.0074x + 0.0011	0.9999	1–250	103.15
G_m_	y = 0.7109x + 0.0077	1.0000	1–1000	95.47
m^1^G	y = 0.1189x + 0.0173	0.9999	1–5000	100.91
C_m_	y = 0.1409x + 0.0316	0.9999	1–5000	102.11
m^5^C	y = 0.7475x + 0.0023	0.9999	1–100	101.46
U_m_	y = 3.8871x – 0.0331	0.9998	1–500	109.75
m^5^U	y = 1.9261x – 0.0852	0.9995	1–250	94.61

## Data Availability

The data presented in this study are available on request from the corresponding author.

## Supplementary Materials

The following supporting information can be downloaded at: https://www.mdpi.com/article/10.3390/metabo12100973/s1. Method S1: Synthesis of stable isotope-labeled 2’-O-methylguanosine, *N*^1^-methylguanosine and 2′-O-methylcytidine. Figure S1: High resolution ESI-MS/MS of (A) 2′-O-methyl-^13^C^15^N_2_-guanosine ([^13^C^15^N_2_]G_m_), (B) *N*^1^-methyl-^13^C^15^N_2_-guanosine ([^13^C^15^N_2_]m^1^G) and (C) 2′-O-methyl-^13^C_5_-cytidine ([^13^C_5_]C_m_). Figure S2: Quantification and statistical analysis results of (A) A_m_, (B) m^6^A, (C) m^1^A, (D) m^6^A_m_, (E) G_m_, (F) m^1^G, (G) C_m_, (H) m^5^C, (I) U_m_ and (J) m^5^U in urine between non-TNBC and TNBC patients. Table S1: The optimized MRM parameters for the analysis of A_m_, m^6^A, m^1^A, m^6^A_m_, G_m_, m^1^G, C_m_, m^5^C, U_m_, and m^5^U. Table S2. Limits of detection (LODs) and limits of quantification (LOQs) of A_m_, m^6^A, m^1^A, m^6^A_m_, G_m_, m^1^G, C_m_, m^5^C, U_m_ and m^5^U in HILIC-MS/MS when malic acid was used or not. Table S3: The intra- and inter-day accuracy and precision for the determination of A_m_, m^6^A, m^1^A, m^6^A_m_, G_m_, m^1^G, C_m_, m^5^C, U_m_ and m^5^U by HILIC-MS/MS method. Table S4: Recoveries of the HILIC-MS/MS method obtained at three different spiking levels. Table S5: Stability of analytes during 72 h at room temperature, 4 °C and −20 °C. Table S6: The normalized levels of methylated nucleosides in the urine samples from NC, EBC and LABC patients. Table S7: The concentrations of methylated nucleosides and creatinine in the urine samples from all participants. Table S8: The cutoff values of A_m_, m^6^A, m^1^A, m^6^A_m_, G_m_, m^1^G, C_m_ and m^5^C. Table S9: Univariate and multivariate analysis of risk factors of early-stage breast cancer.
