# Supplementary material for: Simultaneous Determination of Methylated Nucleosides by HILIC–MS/MS Revealed Their Alterations in Urine from Breast Cancer Patients
Source: Metabolites. 2022 Oct 14;12(10):973. doi: 10.3390/metabo12100973 (PMC9612034; doi:10.3390/metabo12100973)
Supplement: Supplementary file 1 [file metabolites-12-00973-s001.zip › metabolites-1927786-supplementary.pdf]

Supporting Information

for

Simultaneous determination of methylated nucleosides by HILIC-MS/MS revealed their alterations in urine from breast cancer patients

Zhihao Fang[1], Yiqiu Hu[1], Xiujuan Hong[1], Xiaoxiao Zhang[1], Tao Pan[2], Chi Pan[2], Shu Zheng[1,3*], Cheng Guo[1,3*]

[1]Cancer Institute (Key Laboratory of Cancer Prevention and Intervention, China National Ministry of Education), The Second Affiliated Hospital, Zhejiang University School of Medicine, Hangzhou, Zhejiang 310009, China

[2]Department of Breast Surgery, The Second Affiliated Hospital, Zhejiang University School of Medicine, Hangzhou, Zhejiang 310009, China

[3]Cancer Center, Zhejiang University, Hangzhou, Zhejiang 310058, China

[*]Corresponding author:

Shu Zheng, zhengshu@zju.edu.cn

Cheng Guo, cheng_guo@zju.edu.cn

Tel.: +86-571-87784501; Fax: +86-571-87214404.

Table of Content

Method S1. Synthesis of stable isotope-labeled 2′-O-methylguanosine, N^1-methylguanosine and 2′-O-methylcytidine.

Figure S1. High resolution ESI-MS/MS of (A) 2′-O-methyl-$^{13}C^{15}N_2$-guanosine ([$^{13}C^{15}N_2$]G_m), (B) N^1-methyl-$^{13}C^{15}N_2$-guanosine ([$^{13}C^{15}N_2$]m^1G) and (C) 2′-O-methyl-$^{13}C_5$-cytidine ([$^{13}C_5$]C_m).

Figure S2. Quantification and statistical analysis results of (A) A_m, (B) m^6A, (C) m^1A, (D) m^6A_m, (E) G_m, (F) m^1G, (G) C_m, (H) m^5C, (I) U_m, and (J) m^5U in urine between non-TNBC and TNBC patients. (ns $p > 0.05$, * $p < 0.05$, ** $p < 0.001$)

Table S1. The optimized MRM parameters for the analysis of A_m, m^6A, m^1A, m^6A_m, G_m, m^1G, C_m, m^5C, U_m and m^5U.

Table S2. Limits of detection (LODs) and limits of quantification (LOQs) of A_m, m^6A, m^1A, m^6A_m, G_m, m^1G, C_m, m^5C, U_m and m^5U in HILIC-MS/MS when malic acid was used or not.

Table S3. The intra- and inter-day accuracy and precision for the determination of A_m, m^6A, m^1A, m^6A_m, G_m, m^1G, C_m, m^5C, U_m and m^5U by HILIC-MS/MS method.

Table S4. Recoveries of the HILIC-MS/MS method obtained at three different spiking levels.

Table S5. Stability of analytes during 72 h at room temperature, 4 °C and -20°C.

Table S6. The normalized levels of methylated nucleosides in the urine samples from NC, EBC and LABC patients.

Table S7. The concentrations of methylated nucleosides and creatinine in the urine samples from all participants.

Table S8. The cutoff values of A_m, m^6A, m^1A, m^6A_m, G_m, m^1G, C_m and m^5C.

Table S9. Univariate and multivariate analysis of risk factors of early-stage breast cancer.

Method S1. Synthesis of stable isotope-labeled 2′-O-methylguanosine, *N*^{1}-methylguanosine and 2′-O-methylcytidine.

Methylation reactions were carried out by treating a mixture of $^{13}C_5$-guanosine (1 μmol), Me_3SOH (2 μmol), and copper acetylacetonate (1 μmol) in 1 mL dimethylformamide (DMF) at 70 °C for 1 h. Similarly, methylation reactions of $^{13}C_5$-cytidine occurred under the same conditions. The solvent was removed, and the resulting crude mixture was dissolved in water, followed by HPLC purification by using a Waters BEH C18 column (2.1 mm × 100 mm, 1.7 μm). The purified 2′-O-methyl-$^{13}C^{15}N_2$-guanosine ([$^{13}C^{15}N_2$]G_m), *N*^{1}-methyl-$^{13}C^{15}N_2$-guanosine ([$^{13}C^{15}N_2$]m^1G) and 2′-O-methyl-$^{13}C_5$-cytidine ([$^{13}C_5$]C_m) were confirmed by high resolution ESI-MS/MS analysis performed on SCIEX TripleTOF 5600$^+$ mass spectrometer (Figure S1).

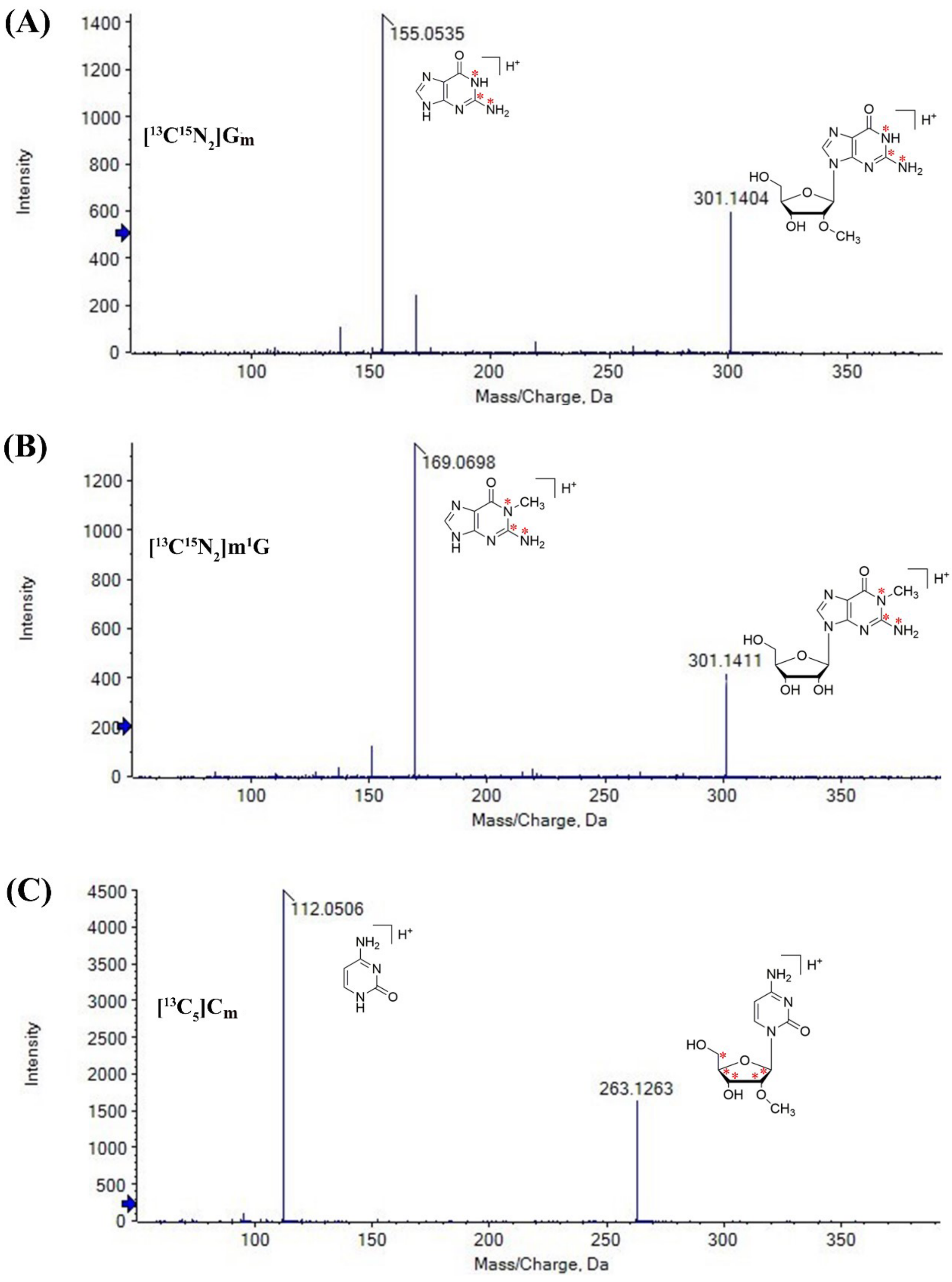

Figure S1. High resolution ESI-MS/MS of **(A)** 2′-O-methyl-$^{13}C^{15}N_2$-guanosine ([$^{13}C^{15}N_2$]G_m), **(B)** N^1-methyl-$^{13}C^{15}N_2$-guanosine ([$^{13}C^{15}N_2$]m^1G) and **(C)** 2′-O-methyl-$^{13}C_5$-cytidine ([$^{13}C_5$]C_m). The theoretical value of $[M + H]^+$ ion of [$^{13}C^{15}N_2$]G_m, [$^{13}C^{15}N_2$]m^1G and [$^{13}C_5$]C_m is *m/z* 301.1404, 301.1411 and 263.1263, respectively. For their product ions, the theoretical value of [$^{13}C^{15}N_2$]$C_5H_6N_5O^+$, [$^{13}C^{15}N_2$]$C_6H_8N_5O^+$ and $C_4H_6N_3O^+$ is 155.0535, 169.0698 and 112.0506, respectively. Asterisk (*) indicates the site of ^{13}C or ^{15}N labeling.

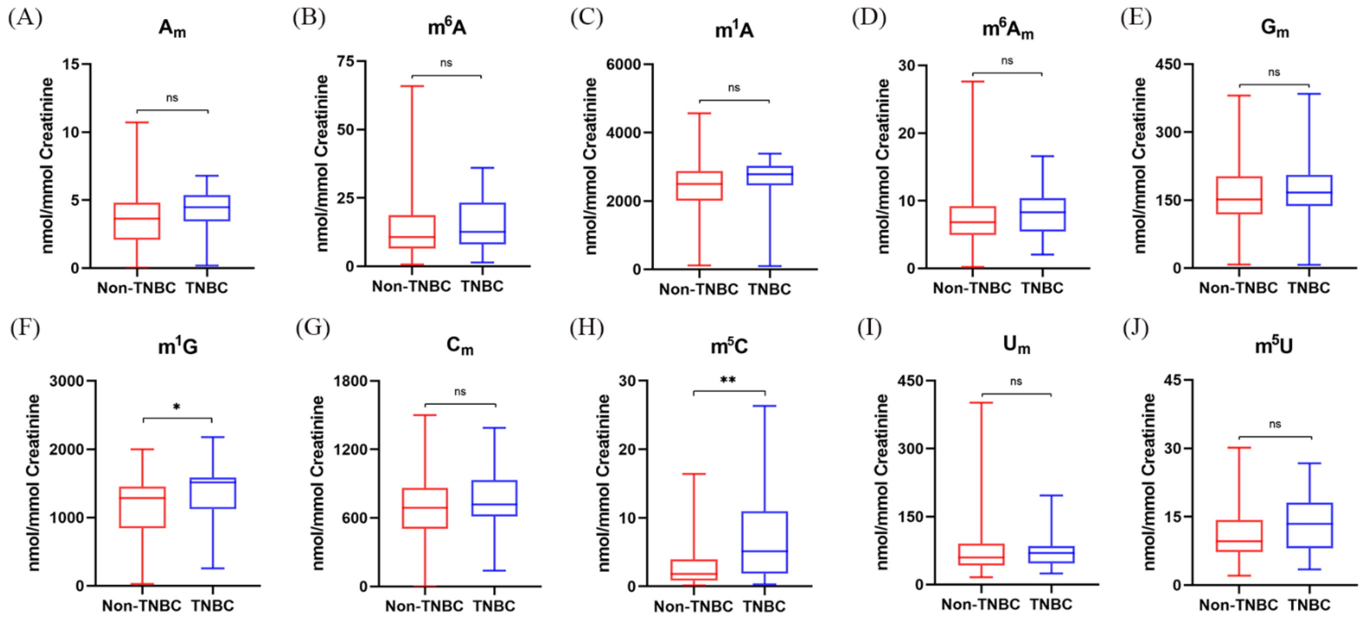

Figure S2. Quantification and statistical analysis results of (A) A_m, (B) m^6A, (C) m^1A, (D) m^6A_m, (E) G_m, (F) m^1G, (G) C_m, (H) m^5C, (I) U_m, and (J) m^5U in urine between non-TNBC and TNBC patients. (ns $p > 0.05$, * $p < 0.05$, ** $p < 0.001$)

Table S1. The optimized MRM parameters for the analysis of A_m, m^6A, m^1A, m^6A_m, G_m, m^1G, C_m, m^5C, U_m and m^5U.

Compound	MRM ion transition (*m/z*)	DP (V)	EP (V)	CE (V)	CXP (V)
A_m	282.1→136.0	50	10	24	10
$[^{13}C_5]A_m$	287.1→136.0	50	12	24	10
m^6A	282.1→150.0	50	12	14	10
$[D_3]m^6A$	285.1→153.0	55	12	14	10
m^1A	282.1→150.0	55	12	14	10
$[D_3]m^1A$	285.1→153.0	55	12	14	10
m^6A_m	296.1→150.0	50	10	25	10
$[D_3]m^6A_m$	299.1→153.0	50	10	25	10
G_m	298.1→152.0	50	8	16	12
$[^{13}C^{15}N_2]G_m$	301.1→155.0	50	8	16	12
m^1G	298.1→166.0	50	8	10	12
$[^{13}C^{15}N_2]m^1G$	301.1→169.0	50	8	18	12
C_m	258.1→112.0	45	10	8	8
$[^{13}C_5]C_m$	263.1→112.0	45	10	16	8
m^5C	258.1→126.0	50	10	16	10
$[^{13}C_5]m^5C$	263.1→126.0	50	10	16	10
U_m	259.1→113.0	45	6	16	16
$[D_3]U_m$	262.1→113.0	45	6	16	16
m^5U	259.1→127.0	55	10	16	14
$[^{13}C_5]m^5U$	264.1→127.0	55	10	16	14

DP declustering potential, *CE* collision energy, *EP* entrance potential, *CXP* collision cell exit potential.

Table S2. Limits of detection (LODs) and limits of quantification (LOQs) of A_m, m^6A, m^1A, m^6A_m, G_m, m^1G, C_m, m^5C, U_m and m^5U in HILIC-MS/MS when malic acid was used or not.

	**LOD**		**LOQ**	
	**without malic acid (fmol)**	**with malic acid (fmol)**	**without malic acid (fmol)**	**with malic acid (fmol)**
A_m	0.25	0.1	1	0.25
m^6A	0.25	0.05	1	0.25
m^1A	0.25	0.05	1	0.1
m^6A_m	0.25	0.05	1	0.25
G_m	0.25	0.05	1	0.1
m^1G	0.5	0.1	2.5	0.5
C_m	0.1	0.05	0.5	0.25
m^5C	0.25	0.05	1	0.5
U_m	1	0.5	5	1
m^5U	2.5	1	10	2.5

Table S3. The intra- and inter-day accuracy and precision for the determination of A_m, m^6A, m^1A, m^6A_m, G_m, m^1G, C_m, m^5C, U_m and m^5U by HILIC-MS/MS method.

QC	Theoretical values (nM)	Intra-day (n = 9)			Inter-day (n = 3)		
		Mean ± SD (nM)	RSD (%)	Accuracy (%)	Mean ± SD (nM)	RSD (%)	Accuracy (%)
A_m	1 (Low)	0.95 ± 0.09	9.62	94.67	0.91 ± 0.01	1.38	90.54
	5 (Medium)	4.82 ± 0.43	8.92	96.41	4.80 ± 0.22	4.50	95.99
	50 (High)	51.09 ± 1.27	2.48	102.19	50.87 ± 1.30	2.55	101.75
m^6A	40 (Low)	36.1 ± 1.1	3.05	90.18	36.5 ± 0.4	1.19	91.28
	400 (Medium)	419.6 ± 13.7	3.27	104.89	417.0 ± 6.4	1.52	104.24
	2000 (High)	1987.7 ± 45.7	2.30	99.38	1996.2 ± 39.1	1.96	99.81
m^1A	200 (Low)	185.0 ± 9.0	4.87	92.50	183.7 ± 7.5	4.10	91.85
	2000 (Medium)	2107.5 ± 98.5	4.67	105.38	2083.9 ± 78.7	3.78	104.19
	6000 (High)	6019 ± 252.9	4.20	100.33	5940 ± 300.0	5.05	99.01
m^6A_m	4 (Low)	4.20 ± 0.10	2.41	104.88	4.22 ± 0.10	2.34	105.62
	20 (Medium)	21.89 ± 0.49	2.25	109.44	21.87 ± 0.48	2.20	109.35
	200 (High)	191.18 ± 4.19	2.19	95.59	191.55 ± 4.03	2.10	95.77
G_m	10 (Low)	9.31 ± 0.37	3.94	93.09	9.52 ± 0.31	3.25	95.22
	100 (Medium)	94.15 ± 3.19	3.39	94.15	94.28 ± 2.35	2.49	94.28
	500 (High)	457.1 ± 20.53	4.49	91.41	467.12 ± 29.00	6.21	93.42
m^1G	100 (Low)	93.5 ± 4.4	4.75	93.46	93.8 ± 3.6	3.80	93.76
	1000 (Medium)	1045.6 ± 19.0	1.82	104.56	1047.5 ± 14.0	1.34	104.75
	5000 (High)	4894.2 ± 74.0	1.51	97.88	4893.1 ± 87.3	1.78	97.86
C_m	50 (Low)	47.5 ± 2.1	4.53	93.01	47.3 ± 1.1	2.24	94.58
	500 (Medium)	543.4 ± 15.1	2.78	108.68	547.1 ± 6.5	1.19	109.42
	2500 (High)	2652.5 ± 46.4	1.75	106.10	2653.7 ± 52.4	1.98	106.15
m^5C	1 (Low)	0.91 ± 0.05	5.55	90.84	0.91 ± 0.06	6.18	90.64
	5 (Medium)	4.75 ± 0.25	5.20	94.93	4.81 ± 0.13	2.77	96.26
	50 (High)	46.06 ± 2.81	6.11	92.12	46.13 ± 3.55	7.69	92.25
U_m	10 (Low)	10.95 ± 0.36	3.30	109.48	10.93 ± 0.53	4.84	109.25
	100 (Medium)	107.63 ± 6.57	6.10	107.63	106.18 ± 7.77	7.31	106.18
	500 (High)	500.9 ± 24.95	4.98	100.19	493.56 ± 28.03	5.68	98.71
m^5U	2 (Low)	2.11 ± 0.13	6.37	105.26	2.08 ± 0.15	7.30	103.90
	20 (Medium)	21.91 ± 0.96	4.40	109.55	21.98 ± 0.56	2.55	109.91
	100 (High)	104.59 ± 8.33	7.96	104.59	101.63 ± 6.99	6.89	101.63

Table S4. Recoveries of the HILIC-MS/MS method obtained at three different spiking levels.

	Added amount (nM)	Mean ± SD (nM)	Average recovery (%)	RSD (%)
A_m	0	7.49 ± 0.21	-	2.85
	1 (Low)	8.50 ± 0.46	101.33	5.45
	5 (Medium)	13.23 ± 0.32	114.75	2.44
	50 (High)	64.34 ± 0.10	113.70	0.16
m^6A	0	488.7 ± 14.7	-	3.01
	20 (Low)	510.5 ± 6.0	108.86	1.18
	200 (Medium)	676.2 ± 11.1	93.75	1.65
	1000 (High)	1482.2 ± 11.3	99.34	0.76
m^1A	0	3382.6 ± 160.9	-	4.76
	100 (Low)	3486.4 ± 36.4	103.86	1.04
	1000 (Medium)	4313.5 ± 177.6	93.09	4.12
	5000 (High)	8434.9 ± 517.1	101.05	6.13
m^6A_m	0	18.51 ± 0.44	-	2.37
	4 (Low)	23.06 ± 0.37	113.72	1.58
	20 (Medium)	40.49 ± 0.74	109.94	1.83
	200 (High)	236.52 ± 6.95	109.00	2.94
G_m	0	138.75 ± 1.50	-	1.08
	10 (Low)	148.34 ± 4.48	95.84	3.02
	100 (Medium)	237.15 ± 7.44	98.40	3.14
	500 (High)	601.56 ± 2.36	92.56	0.39
m^1G	0	1245.5 ± 20.9	-	1.68
	100 (Low)	1349.3 ± 31.4	103.81	2.33
	1000 (Medium)	2264.8 ± 45.8	101.93	2.02
	5000 (High)	6263.5 ± 59.1	100.36	0.94
C_m	0	648.4 ± 5.0	-	0.77
	50 (Low)	695.4 ± 8.6	93.79	1.24
	500 (Medium)	1169.1 ± 6.7	104.13	0.57
	2500 (High)	3256.8 ± 29.6	104.34	0.91
m^5C	0	5.76 ± 0.18	-	3.09
	1 (Low)	6.85 ± 0.49	108.28	7.19
	5 (Medium)	10.84 ± 0.95	101.55	8.77
	50 (High)	58.35 ± 4.70	105.18	8.06
U_m	0	152.42 ± 12.53	-	8.22
	10 (Low)	163.34 ± 9.20	109.21	5.63
	100 (Medium)	247.90 ± 6.19	95.47	2.50
	500 (High)	650.03 ± 8.50	90.51	1.31
m^5U	0	34.81 ± 3.48	-	9.98
	2 (Low)	36.96 ± 1.77	107.87	4.79
	20 (Medium)	60.07 ± 5.81	95.15	9.83
	100 (High)	153.47 ± 9.19	106.44	5.99

Table S5. Stability of analytes during 72 h at room temperature, 4 °C and -20°C.

Compound	Temperature	Room temperature			4°C			-20°C		
	Time (h)	24	48	72	24	48	72	24	48	72
A_m	Low	97[a]	97	98	101	97	97	96	100	97
	Medium	101	98	99	103	104	100	99	99	98
	High	102	97	97	98	99	99	96	101	104
m^6A	Low	100	96	98	101	98	101	96	102	96
	Medium	101	101	98	97	101	102	100	97	98
	High	101	99	96	102	99	99	103	100	103
m^1A	Low	96	97	100	98	103	97	99	102	98
	Medium	101	96	97	101	100	102	102	97	98
	High	99	97	96	103	100	99	97	103	102
m^6A_m	Low	99	100	97	100	96	98	96	103	97
	Medium	100	96	96	100	103	101	99	98	98
	High	99	100	97	101	96	98	100	97	102
G_m	Low	102	96	100	98	99	97	98	99	96
	Medium	98	97	97	99	101	102	97	99	98
	High	99	97	97	103	100	98	99	101	101
m^1G	Low	99	97	103	98	96	98	98	101	98
	Medium	100	96	100	99	101	102	97	98	97
	High	100	97	97	102	100	100	99	103	103
C_m	Low	99	100	99	102	98	100	98	104	96
	Medium	100	101	100	99	103	103	103	101	101
	High	100	96	96	100	97	98	99	102	101
m^5C	Low	97	101	103	101	97	102	96	101	103
	Medium	102	99	103	101	103	103	99	101	99
	High	98	97	97	100	101	98	99	102	102
U_m	Low	102	100	100	103	101	97	98	98	98
	Medium	100	104	98	101	104	99	100	101	102
	High	99	100	103	103	104	98	98	103	104
m^5U	Low	100	103	100	97	101	98	100	102	101
	Medium	101	98	99	104	98	102	103	96	104
	High	96	99	97	102	101	98	102	101	99

[a] All values were expressed as percentages, and the concentration of fresh samples at 0 h were considered as 100%.

Table S6. The normalized levels of methylated nucleosides in the urine samples from NC, EBC and LABC patients.

		A_m	m^6A	m^1A	m^6A_m	G_m	m^1G	C_m	m^5C	U_m	m^5U
NC	Mean	4.95[a]	29.67	2840	8.55	204.8	1458	793.2	5.12	67.41	9.86
	SD	0.24	5.16	96	0.48	11.3	50	33.8	0.49	6.32	0.53
EBC	Mean	3.27	27.81	2373	7.30	160.8	1109	674.4	3.17	79.15	11.29
	SD	0.17	8.40	77	0.36	6.5	45	25.1	0.30	7.11	0.57
LABC	Mean	4.52	45.31	2514	8.61	196.1	1320	804.2	4.40	92.12	13.05
	SD	0.58	18.33	103	0.51	11.2	63	37.1	0.61	8.71	1.28

[a]nmol/mmol Cr

SD: standard deviation, NC: normal control, EBC: early-stage breast cancer, LABC: locally advanced breast cancer

Table S7. The concentrations of methylated nucleosides and creatinine in the urine samples from all participants. (ND: Not Detected, B: Breast Cancer, N: Normal)

No.	Cr (μM)	A_m (nM)	m^6A (nM)	m^6A_m (nM)	m^1A (nM)	G_m (nM)	m^1G (nM)	C_m (nM)	m^5C (nM)	U_m (nM)	m^5U (nM)
B1	18414	73.2±3.9	254.4±14.3	109.7±0.9	43052±210	2028±25	23687±209	11832±531	39±2.4	637.3±21	100.5±2.3
B2	8277	45.9±0.6	429.6±2	80.2±1.8	17526±1207	1224±30	9999±36	4627±54	7.7±0.4	348.5±16.8	117.7±9.7
B3	4272	13.7±1	19.9±1.8	28.4±0	6195±544	506±20	127±2	1859±16	19.1±1.9	184.2±16.4	93±6.2
B4	15502	54.2±3.7	708.2±10	76.2±0.1	26081±39	2293±12	13142±128	8851±15	23.8±2.2	701.1±58.7	209.6±4.1
B5	3341	13.7±0.7	147.5±7.7	26.6±0.1	6399±114	529±4	3053±70	1899±6	9.6±0.2	171.3±15	32.2±2.6
B6	13872	44.7±2.8	202±2.2	70.5±2.3	28747±59	1877±36	14441±199	6271±74	5.6±0.4	334.6±15.6	111.5±6.4
B7	17088	47.9±2.2	54.6±4.7	109±1.5	39229±310	2232±43	18596±352	9332±299	8.5±0.4	3114.1±115.4	233±15.6
B8	8973	55.9±1.1	12.8±0.2	133.9±1	5661±199	2706±28	ND	10908±162	38±3.5	706±36.6	87±3.6
B9	6696	18.9±0.3	16.7±0.8	37.7±0.7	5944±291	861±4	ND	3180±25	36.9±1.6	361±3	66.6±1.3
B10	3706	1.9±0.1	12.9±0.8	38.3±0.3	ND	155±11	ND	3118±18	2.5±0.1	672.2±46.7	48.7±9.6
B11	8537	20.8±0.5	5.2±0.1	43.2±0.6	ND	900±40	ND	3505±76	6±0.5	322.6±23.1	34±0.6
B12	6261	2.6±0.2	54.6±1.5	26.1±0.8	2871±161	835±13	5170±147	3166±118	9.8±0.3	359.7±23.5	78.7±7.3
B13	1563	8±0.5	2485.4±6.2	17.5±0.5	2082±46	320±3	2217±58	1779±1	12.6±1.1	192.7±3.5	163.1±6.2
B14	19447	89.6±4.1	400±18.7	118.4±2.5	49366±1280	3161±68	29437±557	16466±116	11.1±0.1	1091.7±16.3	193.4±7.9
B15	11093	63.3±2.3	909.1±17.7	93.5±3.2	32972±455	2515±10	19600±578	8401±360	14.1±1.2	728.6±36.5	148.5±0.3
B16	2702	9.4±0.6	6.9±0.3	30.1±1	ND	428±4	ND	2070±22	3.5±0.2	288.3±25	41.6±0.7
B17	3154	13.7±1.1	25.6±1.7	24.4±0.1	7398±22	469±13	543±12	1512±5	9.3±0.5	149.9±5.8	80.5±5.9
B18	3941	0.6±0	63.5±1.9	0.9±0	ND	31±2	123±2	716±45	2.4±0.2	135.6±1	16.2±1
B19	11958	63.5±2.8	1011.6±13.7	111.8±4.7	25092±1244	2419±23	13994±93	8745±23	4.2±0.2	619.8±20.4	98.8±3.5
B20	17880	43.3±3.7	13.3±0.8	141.2±10.5	3642±244	2497±8	ND	11147±8	45.5±0.5	734±29.9	58.7±1.6
B21	20983	66.8±5.8	803.7±20.6	140.4±3.1	40023±623	1821±1	21903±138	12043±155	29±0	1039.4±92.1	178.7±0.9
B22	11202	50.1±0.7	408.1±1.5	112.1±0.1	20808±149	2681±16	13988±39	11115±147	11.5±0.3	806.6±5	165.4±12
B23	13313	38.6±1.1	20±1.2	98.2±0	9087±590	2336±34	ND	9323±256	8.7±0.8	426.6±32.5	75.7±3.4
B24	23251	173.6±0.6	437.8±1.8	343.1±31.9	56220±3590	6719±93	18124±535	28417±566	46±3.3	2619.6±202.2	291.1±11.4
B25	6807	25±0.4	131.9±0.3	50.2±0.2	20747±576	1212±24	10193±65	5618±74	106.5±7	582.7±58.9	96.8±5.8
B26	12929	54±2.6	270.2±7.7	73±0.8	39548±1624	1959±42	18670±16	8071±28	29.4±2.1	413±8	125.3±3.2
B27	4051	19.7±1.7	96.2±0.1	21.1±0.3	11702±96	632±8	5575±21	2358±23	12.4±0.5	127.6±7.4	33.3±1.4
B28	7169	35±2.6	3110.4±102.5	73.4±1.6	13482±306	1873±10	9819±54	5282±2	19.8±1.7	369.6±18.5	105.4±0.1
B29	5716	9.7±0.1	6.9±0.1	47.8±1.1	ND	931±0	ND	2998±23	3.2±0.2	289.3±5.7	49±1.2
B30	5459	13.4±0.6	90.8±1	58.5±0.5	9835±225	1342±21	2337±5	2461±78	5.9±0.4	1127.8±48.7	53.9±0.4
B31	4408	17±0.6	4359.2±114.4	49±1.6	2618±216	1063±9	2465±75	3674±39	7.6±0.6	230.7±10.8	68.6±6.2

B32	5213	20.7±1.1	6.2±0	31.4±0.8	19±1	634±11	ND	2825±12	9.3±0.2	352±6.3	40.6±2.3
B33	4906	0.7±0	44.9±1.6	18±0.4	ND	484±8	118±20	213±2	12.5±1	1353.2±106.4	33.2±1.4
B34	7253	13.4±0	414.7±28.2	55.1±0.3	3825±121	705±1	1919±17	3989±109	11.4±0.3	418.9±12.5	74.9±7.1
B35	11272	2.3±0	7.7±0.3	53.3±1.5	ND	1476±1	ND	6035±152	7.4±0.6	723.4±29.1	30.7±2.9
B36	19549	55±1.7	107±2.9	91±0.1	48836±1681	2260±3	27161±80	12858±121	47.7±2.8	1077.8±6.6	192.7±15.2
B37	18066	94.9±6.8	168.2±0.6	236.8±2.8	46640±718	6373±99	24954±777	21844±155	9.9±0.9	1519.6±97.3	173.8±13.6
B38	7158	15.8±0.4	64.8±2.2	16.3±0.2	14178±664	724±15	5843±45	3880±125	78.6±2.7	249.4±17.7	96.1±9.2
B39	21145	56.1±1.7	134.2±7.2	105.6±3	49627±901	2749±23	26211±193	12219±178	59.9±3.2	344.5±9.3	181.5±3.2
B40	3979	0.7±0	22.7±2.3	22±0.1	ND	26±2	ND	2593±7	20.8±0.7	321.7±29.1	58.2±1.2
B41	1952	12.9±0.4	30.6±1	28±0.7	6590±384	552±3	3100±28	1789±14	9.9±0.4	187.6±16.5	52.2±2.9
B42	6050	15±0.3	382.1±14.8	34.2±1.4	14413±802	582±18	487±6	3208±31	69.5±4.7	252.6±3.6	68.3±3
B43	4061	16.8±0.7	91.9±1	21.1±0.2	12004±162	586±18	5910±184	2130±43	31.2±2.7	202.4±8.6	49.8±4.8
B44	4093	1.4±0.1	218.9±2.5	10.3±0.2	4049±74	784±3	3441±21	2553±37	5.3±0.3	184.9±7.4	31.1±1.1
B45	20549	90.8±0.1	252.4±3.5	100±3.1	57313±770	3399±95	32146±165	15044±126	84.6±6.1	862.6±0.6	188.3±11.3
B46	8006	8.5±0.3	11.4±0.2	43.5±2.3	770±15	796±25	ND	4377±35	3±0.1	618.2±43.8	27.4±0.9
B47	5041	30.4±1.2	588.2±5.8	72.4±1.9	10858±82	1250±17	6936±13	4915±129	35.8±2.1	433.5±0.8	88.4±6.6
B48	8601	20.2±0.2	7.4±0.4	39.7±0.7	1627±14	827±11	ND	3590±57	5.2±0	361.7±2.8	122.3±9.8
B49	24600	89.5±0	787.9±17	178.2±5.1	46656±2052	3904±42	23553±252	17297±58	14.1±0.3	1528.7±46.5	212.6±14.8
B50	9298	51.9±4.7	16.1±1.1	53.1±2.5	10434±560	1292±56	ND	6443±36	23.7±0.4	283.1±20	73.6±6.8
B51	9034	44.7±1	777.7±14	89.6±0.3	26150±36	1553±11	12283±7	5094±53	5±0.5	338.7±4.1	122.9±8.9
B52	17126	2.1±0.1	157.6±1	89.8±0.5	8977±445	930±19	3275±31	8182±114	8.2±0.4	1204.6±4.2	66.8±5
B53	14269	75.2±1.3	265.3±5.6	176±2	31962±2743	3116±5	18452±255	13080±135	15.9±0.1	1144.3±74.2	151.3±5.3
B54	14633	36.6±3.2	15±0.7	89.6±1.4	11936±174	1231±3	ND	7824±52	3.5±0.3	893.9±17.5	38.2±3.4
B55	2061	7.5±0.4	6.3±0.4	13.1±0.1	240±6	247±3	ND	836±35	3.4±0.2	80.5±6.3	48.3±2.8
B56	12714	50.9±4.4	299.7±6.3	66.9±1.4	23772±595	1600±23	15697±28	7864±22	3.7±0.3	698.2±44.4	219±21
B57	12815	52±1.5	527.6±17.4	84.2±2.4	27556±859	2237±18	10348±129	8809±157	8.6±0.4	674.7±52.8	191.1±12
B58	18887	110.7±1.4	648.4±8.2	173.9±0.3	38069±2036	3831±79	24142±150	18517±10	11.1±0.1	1054.9±46.2	187.3±13.9
B59	9263	37.9±1.1	81.2±1.7	58.1±1.8	25516±748	1268±28	1004±28	6119±47	10.4±0	720.1±14	185.9±14.5
B60	5784	20.3±0.5	87.1±1.1	27.7±0.7	17254±266	565±27	8153±52	2410±71	6.1±0.6	202.9±16.5	87.3±6
B61	12716	57.3±1.4	1127.1±37.2	141.3±1.3	28100±1885	2965±55	16864±339	11576±119	132.2±12	1238.2±18.8	157.3±2.8
B62	3756	22.1±0.7	74.6±1.9	39.2±0.1	11255±324	879±28	2492±5	2998±27	7.6±0.6	261.2±12.7	49.8±4.8
B63	17627	44.9±1.1	207.3±2.2	125.6±3.6	37443±454	2380±80	529±8	10067±334	16.9±0.1	607.2±24	129.5±5.1
B64	24505	185.2±5.9	240.9±6.2	352±33.1	66640±3231	8137±47	32078±345	28751±47	10±0.8	2994.1±165.5	184.1±17.3

B65	17312	69.3±1.7	1140.4±16.4	118.8±2.5	41991±67	3483±34	23006±609	18141±18	5.1±0.2	1031.8±56.1	120±5.8
B66	7429	41.8±0	361.8±2.9	54.6±0.1	22253±777	1296±12	10439±114	5221±62	6.6±0.5	366±12.2	95.2±8.6
B67	18694	58.5±0.3	1176.2±30.9	117.4±4.9	46713±1910	2700±8	30507±397	11012±23	28.2±2.4	623.2±19.6	73.1±7.1
B68	14676	49.9±2.5	125.4±6.8	115.7±3.2	39685±2560	2432±15	1469±18	13444±13	12.1±0.6	1188.9±98.6	187.2±16.7
B69	1669	4.5±0.4	772.5±22.7	11.7±0.2	2962±70	230±1	2043±29	776±25	1.6±0.1	96.9±3.4	28.1±1
B70	4093	17.2±0.6	1426.7±17.6	62.4±0.3	13843±558	1528±22	8905±4	5180±140	107.8±9.2	698.9±42.7	189.7±13.5
B71	14757	23.3±0.8	333.9±0.5	78.4±0.5	33362±2435	2364±24	20305±835	12350±587	18.7±1.7	902.8±24.5	156.7±15.6
B72	6353	29.5±1.7	614.4±9.8	52±0.5	19546±177	1271±50	12880±254	5090±48	14.1±1.3	337.3±9.7	129.5±2.2
B73	22362	99.4±0.3	2100±49.9	150.9±2.2	59953±963	4925±40	39923±188	19904±52	127.4±1.5	1640.2±146.6	203.1±13.9
B74	8243	427.8±1.1	135.2±2.7	44.8±0.3	15361±308	2197±12	10578±8	8806±179	82.3±2.8	1045.2±96.8	248.6±17.1
B75	15017	54.6±0.3	353.7±8.6	75.7±1.2	38745±3080	2210±15	22736±344	11417±98	20.8±0.1	744.1±9.5	166.2±7.3
B76	15496	60.3±1.9	542.8±21.6	94.6±4.4	32798±18	1893±11	17424±93	6481±76	8.4±0.3	452±18.2	73.4±1.2
B77	8054	30.6±2.5	116.4±7.6	39.4±1.5	21912±1261	943±16	10193±151	4398±55	4.7±0.3	267.5±7.9	51.7±4.8
B78	22965	179.6±0.6	401.3±6.9	634.4±10.9	48297±143	13328±430	31563±652	39525±190	3.9±0	7513.9±612.2	215.7±2.4
B79	7135	36.4±2.4	202.9±11.1	80.4±1	21166±729	1612±16	11624±354	6681±17	47.6±3.8	592±30.3	141±2.2
B80	14767	29.9±0.9	211.5±6.7	60.7±0.5	23547±676	1893±24	1701±38	11268±179	49.1±2.8	899.1±32	310.3±27.1
B81	11065	59.3±0.9	223.6±5.4	130.3±0.4	33774±1554	2968±41	2479±13	10695±36	19.8±1.3	569.1±21.6	232.4±2.9
B82	23535	73.9±5.9	198±8.3	203.2±0.3	46755±969	5221±57	ND	22127±119	139.4±3.8	2600.5±258.1	129.6±10.3
B83	3368	14.3±0.9	18.3±0.5	17.5±0.2	9596±208	536±6	146±2	2593±6	15.2±1.5	324.2±24.3	150.1±8.5
B84	9496	18±1	8.4±0.5	29.9±0.3	386±7	1861±26	2232±14	7652±137	1.4±0	595.8±3.3	23.7±1
B85	17053	101.1±2.4	172.2±4.4	120.8±0.3	26280±450	3453±33	18561±64	16597±204	10.4±0.8	1344.3±123.6	223.9±9.9
B86	12063	54.5±3.5	187.9±5	66.7±2.8	32911±2449	1256±16	8727±23	7449±43	14.3±0.8	409.8±29.6	112.3±5.8
B87	12155	31.6±1.8	9.2±0.6	77.7±6.2	1859±11	1415±19	ND	9780±148	30.4±1.1	506.6±8.6	37.5±0.2
B88	10484	17.8±0.1	6.9±0.1	116.2±8.3	ND	1519±2	ND	8801±112	13.6±0.7	712.1±76.1	184.3±12.3
B89	14615	62.6±6	291.6±8.8	99.2±2.1	38760±1856	3245±5	24851±181	15286±55	44.7±0.7	714.4±29.7	188.5±18.5
B90	4867	1.7±0.1	193.7±0.9	47.9±0.7	6160±157	325±2	1875±59	3723±59	5.7±0.5	438±24.3	92.2±0.8
B91	16474	2±0.1	10.9±0.6	113.5±7.1	ND	1923±47	ND	12822±118	15.9±0.4	1198.8±76.6	64.1±1.2
B92	15752	9.2±0.3	363.7±4	31.8±0	31988±355	1805±16	3968±48	7620±76	27.7±0.6	480.2±12.4	118±4.3
B93	21083	12.2±0.4	131.3±4.3	59.6±2.2	31282±1654	3826±163	11282±76	12868±19	4±0.1	2826.4±248.4	534.1±47.6
B94	10182	40.5±0.1	338±5.2	93.9±3.9	27768±846	2753±74	6301±82	10195±156	93.4±5.1	1437.1±11.4	159.3±8
B95	17678	65.9±0.7	556±15.5	108.9±5	41484±954	2218±16	24764±211	13607±15	37.1±0.4	1319.6±1.3	237.4±15.8
B96	4365	10.7±0.4	32.4±0.2	21±1.4	10509±494	499±4	4810±37	1957±38	5.9±0.3	225.3±9.8	67.2±2
B97	12579	8.7±0.3	80.5±0.1	54.3±0.1	28572±1195	1654±9	8947±31	914±2	6.9±0.6	4220.3±244.3	57.1±5.1

B98	7633	2.1±0.2	33.1±1.5	16.8±0.1	23013±1004	579±5	5643±125	2395±9	2.4±0.1	3056.9±213.4	33.4±0.9
B99	11398	2.1±0	29.8±0.1	3.8±0.2	45695±1027	2573±51	ND	1884±10	5±0.3	3429.4±194.8	64.5±3.6
B100	13828	13.5±0.4	48.1±1.1	38.1±0.9	34879±1601	1629±25	8008±284	5715±39	10±0	2285.1±74.9	86±6.6
B101	20483	119.2±0.4	295.8±6.8	200.6±6.5	50853±912	4042±60	28889±407	21550±138	134.9±1.5	1121.4±16.3	128.1±2.8
B102	6073	21.2±0	34.7±0.7	40.5±1.9	15845±439	871±20	5637±52	2195±9	6±0.4	957.7±78.4	94.2±4.4
B103	6341	1.9±0	151.4±1.6	61.8±1.3	19116±450	1346±53	8860±102	4952±84	16.7±0.5	305.3±0.6	83.6±3.6
B104	10743	1.9±0.2	108.2±1.5	77.2±2.1	37418±676	2146±11	9941±203	10223±308	17±1.2	939±19.2	177.9±8.1
B105	23788	81.9±1.5	210.2±4.5	115.9±1.6	55129±1302	3692±67	31424±115	16440±281	18.1±0.4	806.8±11.9	122.9±2.9
B106	9635	52.5±3.2	67.5±0.2	80.8±1.7	25997±108	1305±2	15291±284	7371±184	14.6±0.8	601.8±30.6	91.2±6.5
B107	10377	33.3±0.9	231.5±4.2	127.1±0.2	33345±2973	2817±33	15524±113	12707±133	24±0.9	1317±109.7	160.8±0.7
B108	22286	111.4±1.1	196.6±3.3	176.2±16	59227±507	4168±26	33721±816	18127±352	31.6±0.7	1825.4±90.3	197.3±2.5
B109	14836	48.6±1.5	174.8±1.4	114.9±3	32855±1755	2265±17	18526±178	7579±146	13.3±0	475.9±34	140±9.2
B110	9223	34.2±3.3	96.5±2.7	84±2	25452±312	1390±22	12286±310	8387±59	151.3±9.2	789.3±40.9	164.6±14.3
B111	6339	26±0.4	53.1±0.2	53.3±0.2	17587±324	897±4	9614±197	4489±20	102.3±9.3	428.4±34.7	78.2±7.5
B112	7199	25.9±2.2	60.3±3.2	28.8±0.2	18969±572	671±9	9679±58	3453±125	21.2±2.1	196.7±15.7	63.9±1.4
B113	5571	17.5±0.4	564.2±9.8	27.9±0	11746±1220	632±14	6343±80	3431±67	69.3±3.5	391.9±1.2	80±5.8
B114	29124	214.3±8.8	520.4±5.8	624±17.5	75289±4851	13841±347	43764±107	50229±277	198.4±4.4	5022.5±92.8	280.8±26.3
B115	3542	1±0	30.6±2.3	29.1±0.2	3569±29	875±0	2756±131	3421±147	6.7±0.1	353.2±19	25.5±1
B116	4290	0.8±0	21.4±1.9	7.9±0.1	13796±1437	521±26	6383±18	109±2	2.2±0.2	1140.6±26.4	21.3±0.9
B117	19477	30.1±0.3	258.8±11.5	80.9±3.3	46586±478	3120±46	25577±551	14625±76	54.4±5.1	1659.4±41.4	132.4±1.2
B118	17077	61.4±6	127.7±5.4	121.5±1.7	43713±2241	3566±114	20421±59	16103±8	41.4±3.5	877.9±37	146.3±7.6
B119	14778	91.6±9.2	234.4±4.3	373.9±12.7	29235±1466	9042±329	18071±62	20864±106	54.7±1.6	3283.4±151.3	117.2±4.2
B120	11234	50.3±0.6	117.5±0.2	90.7±0	30058±991	1692±12	16443±150	7126±113	117.4±10.4	670.4±25.2	122.1±4.7
B121	15504	83.5±4.6	274.8±5.7	140.4±6.8	59827±833	3076±80	26462±119	12509±64	28.9±2.4	875.8±5.1	222.5±5.2
B122	28686	119.7±11	290.1±3.1	127.8±5.8	72342±2314	3915±83	39132±445	19468±864	153.6±15.1	1209.3±16.1	114.7±7.4
B123	26275	61±2.2	255.2±6.2	133±4.4	52218±257	2654±10	33678±548	15658±10	87.5±6.1	843.8±8.4	201.4±18
B124	3417	1.3±0.3	25.7±1.4	28.9±0.5	6724±311	1243±58	16±0	ND	2.6±0.2	2105.7±171.3	14.2±0.8
B125	12196	115.2±0.2	137.3±10.2	222.6±4.8	33693±1261	4336±88	17648±287	18290±204	391.5±10	2598.1±23.7	272±25.4
B126	16025	60.2±0.5	214.6±10.8	239.4±0.6	38356±2449	4374±69	19197±67	15064±454	66±4.2	1467.4±18.1	211.3±19.2
B127	19432	95.7±4.6	698.2±6.4	190.6±10.6	52462±3202	3754±49	29413±476	15710±24	239.3±21	1656.8±97.6	200.4±6.8
B128	10268	43±0.3	49.3±0.6	60.5±2.6	25367±263	1634±4	12939±378	6713±66	14.4±1	915.3±33.3	142.7±13.3
B129	25654	120.4±3.8	276±6.1	216.7±3	54922±1239	4600±1	32628±479	19821±348	64.5±0.8	1225.3±115.8	201.4±9.8
B130	6307	13.8±1.1	94.5±1.7	20.2±0.1	22097±327	800±17	8522±90	3295±42	21.4±0.5	284.4±10.3	119.5±11.6

B131	22310	200±11.5	222.5±11.8	327.2±0.4	65481±1596	8476±428	41860±442	37304±183	137.7±3.9	3955.8±325.1	169.2±1
B132	6554	27.8±1.4	63.7±4.4	68.6±0.3	18195±152	1154±17	7348±159	6711±106	30.7±1.3	355.9±2.7	81.7±1.6
B133	16059	53.1±3.3	90±0.7	57.8±0.5	41178±525	1519±9	17008±58	4496±0	8.9±0.1	971.2±82.5	69.9±0.5
B134	4182	4.2±0.2	46.3±1.9	18.2±0.2	9594±444	184±9	1155±53	956±7	3.2±0	506.2±28.6	16.9±1.8
B135	20318	100.3±1.2	209.4±5	128±2.1	53375±108	2916±52	31997±445	14849±246	199±11.8	939±15.7	164.3±10.6
B136	27269	46.2±4.1	47.5±4.2	78.2±2.2	26907±671	2062±57	16591±502	8498±18	5.9±0.1	936±51	196.8±20.1
B137	10606	18.4±1.6	26.2±1.6	32.4±1.9	17739±78	499±2	8058±40	2602±107	2.4±0.2	581.5±13.6	34.7±3.5
B138	10696	15.3±1	174±1.1	35.6±0.3	11770±1073	878±34	5240±138	2933±40	4.5±0.4	248.1±16.8	81.1±9.3
B139	6853	49.4±0.4	125.7±4.2	90.3±0.2	31295±150	1427±33	15030±313	6769±41	33.2±0.3	632.8±29.1	135.9±9.3
B140	10940	18.5±1.5	485.3±17	46.6±0.1	27884±925	1529±50	11759±293	5502±2	41.3±4.1	221.6±1.5	96.6±6.8
B141	11932	4.7±0	148±6.5	88.8±2.6	19622±278	2301±6	10054±58	8216±146	59.8±4.2	349.8±2.2	98.8±5.5
B142	3631	17.5±0.3	68.2±3.3	79.1±3.3	24637±667	1172±24	11594±45	3083±87	1.8±0.1	2520.3±192	69.7±7.5
B143	9442	40.5±0.5	124±2.5	59.5±3.4	29250±465	1748±52	14294±119	8130±231	76.1±4.6	908.6±94.7	272±19.5
B144	8711	93.2±5.6	227.4±0.4	128.3±1.9	70868±240	4736±76	44535±452	18543±575	56.4±4.5	699.4±3.2	208.3±1.8
B145	4173	11.6±0.1	52±0.3	22.1±0.6	8576±626	463±12	4082±68	1887±35	26.1±2.3	139.2±10.4	76.1±0.9
B146	6436	5.2±0.2	171.6±4.8	46.2±0.2	14929±11	971±13	6841±155	4552±38	8.1±0.6	482.3±16.5	50.6±3.6
B147	9134	19.3±1.2	76.6±2.5	31.3±0.9	17123±1	514±5	7090±114	3344±44	24.9±2.5	201.7±6.1	43.3±3
B148	6338	38.3±2.2	127.9±0.3	48±0.1	17057±1334	1030±18	9530±54	3883±115	51.2±3.5	274.1±14.6	139.5±13.6
B149	7869	28.3±2.8	123.4±1.9	40±1.4	16297±662	923±4	8997±214	3635±32	34±1.5	262.7±17.2	65.3±4.8
B150	12667	58.5±2.4	150.7±3.2	222.1±3.9	38601±1094	4255±93	18126±628	14061±176	33.9±3.1	1665±9.1	111.9±4.6
B151	8485	21±1.8	56.4±1.4	41.3±2.2	17985±1020	1028±29	8764±196	4074±107	35±2.7	326.1±27.5	46.7±3.1
B152	6929	19.9±0.1	64.1±0.7	28.4±0.5	18273±335	585±8	9200±74	3125±19	4.3±0.2	284.6±18.8	47.8±1.6
B153	9122	41.1±1.2	110.4±4.5	72.7±1.5	21568±127	1414±19	12616±173	6727±129	121.2±7.5	648.3±55.6	134.4±4.4
B154	4799	0.1±0.2	18.1±1	13.8±0.7	11156±707	480±10	3716±64	891±9	5.2±0.5	507.4±28.5	17.9±0.5
B155	3390	16.3±0.6	33.3±1.7	31±0.4	6950±143	668±15	3511±42	2454±95	20.7±0.3	293.2±7.5	66.4±5.8
B156	1357	10.6±0.8	44.9±3.5	20.5±0.7	5087±533	341±1	2709±63	1808±19	13.8±1.3	124.8±8.5	27.1±2.8
B157	3885	2.7±0	18±1.7	27.2±0.4	6960±509	660±10	2916±88	1781±57	3.3±0.3	360.1±6.7	30.7±3.4
B158	8063	2.7±0	155.1±1.5	24.6±1	16605±750	984±17	9171±86	3453±8	5.6±0.4	316.5±2.5	108.4±10.5
B159	6590	0.5±0.2	30.9±1.2	17.3±0.6	11859±343	638±24	1713±15	1970±21	2.3±0.2	247.2±24.9	48.1±1.2
B160	3844	3.4±0.1	18.3±0.6	17.9±0.3	10758±260	515±2	5267±267	1707±18	2.9±0.3	400.2±22.3	31.4±3.4
B161	4707	28.7±0.7	82.6±6.1	78.2±0.4	14970±311	1724±1	7043±54	6431±54	7.5±0.2	701.4±20.8	96.7±7.5
B162	13898	32.9±0	137.5±2.1	51.3±2.4	23749±1888	955±13	12041±44	4786±82	9.7±0.6	364.9±6.3	75.9±7.7
B163	18232	56.2±0.4	223.1±4.6	101.8±1	49989±1527	1532±5	26008±510	11846±298	120.9±0.2	1022±4	171.9±11.5

B164	10779	50.7±1.9	146.7±2	68.8±1.7	33823±2177	1933±28	17378±133	8446±84	121.4±3.1	752.2±37.3	93±8.8
B165	7633	68.7±5.3	93.4±3.2	111.8±4.2	28764±884	2125±54	14307±209	8226±58	69.6±8.2	639.8±27.8	197.2±12
B166	19409	106.7±4.9	199.2±15.6	113.8±0.3	64798±604	3282±28	37291±303	14933±92	20.3±1.5	1154.8±74.5	77.7±8.7
B167	4808	9±0.2	41.5±2	54.4±0.3	15397±1269	459±7	6217±75	3730±36	5.3±0	577±17.6	28.5±3.6
B168	3148	14.7±0.4	23.3±1.3	19.1±0.1	8938±211	290±2	4741±23	1487±9	11.1±0.6	104.4±9.2	34.2±4.1
B169	10131	43.1±0.8	71.8±1	68.9±0.3	23666±674	1424±20	10845±5	6026±96	31.1±1.8	412.8±33.6	114.3±9.9
B170	7598	26.8±1.7	49±1.2	26.7±0.2	22031±880	951±4	11351±414	3169±51	14.4±1	138.7±11	74.7±8.5
B171	14917	60.5±4.6	195.5±4.7	116.4±1.8	38353±209	2022±92	21965±132	12988±287	47.3±4.4	753.9±16.1	156.2±4.2
B172	22203	101.5±1	592±19.8	237.7±9.6	50672±1521	5046±77	29024±837	20592±757	54.2±5	1380.3±56.2	223.4±11.8
B173	4672	13.2±0.4	101.1±6.6	22.5±0.3	13424±475	619±1	6074±86	2698±38	41.9±2.4	262.3±24.8	87.6±6.8
B174	3569	12.9±0.3	31.9±3	20.4±0.4	8418±343	537±16	1740±48	2058±22	4.7±0.4	271±6.3	36.3±1.7
B175	5327	4.2±0.1	25.7±1	14.2±0.1	14280±321	395±1	5182±2	2049±30	5.1±0.5	301.5±22.9	43±3.2
B176	13112	5.5±0.3	295.2±7.7	75.7±4.7	29308±1251	1504±11	11889±47	7367±185	3±0.2	783.1±62.9	113.5±8.3
B177	4122	20.9±0.7	73.8±0.2	50.2±0.4	11873±562	843±31	5774±148	3115±81	8±0.5	233.3±2.1	82.2±6.9
B178	3225	26.2±0.4	51.9±1.1	85.6±1.7	10657±916	1632±1	5303±12	6503±228	10.5±0.3	652±46.9	50.9±5.8
B179	12403	45.3±2.7	62±2	50.7±1	31548±530	1536±1	13386±440	7245±109	23.3±0.8	340.1±32.5	60.9±4.8
B180	16243	17.3±1.3	76.4±4.2	84.2±1.9	44315±1324	2850±83	24552±158	12489±118	29.7±2.7	1181.4±106.5	35.3±2.4
B181	11600	3.4±0	482.4±26.7	118.6±2.8	35979±1532	2569±69	7189±184	10438±252	13.4±0.9	1059.1±94.2	92±4.8
B182	9263	74.7±3.8	187.1±6.6	196.4±0.1	24882±923	3283±46	13825±224	9931±258	10.7±0.3	1110.3±54.5	124.4±5.7
B183	15867	50.3±1.9	29±2.2	120.4±2.3	44300±1913	2101±72	28±9	9872±159	37.5±3.1	441.1±23.7	32.6±0.1
B184	10334	52.6±4	116.6±2.8	101.4±2.6	27292±312	2146±13	16095±43	10618±121	109.7±9.8	1130.7±46.2	94.3±1.5
B185	3737	22.2±0.1	119.1±5.4	37.8±1.4	11420±1312	611±3	4375±203	2405±23	14.8±0.8	172.5±10.4	51.8±5
B186	5444	21.8±1.3	43.6±1	47.5±3.9	15946±171	1084±12	9103±116	5732±134	81.5±8	592.2±16.8	69±3
B187	12342	16.4±0.6	61.1±4.9	82.7±2.1	1489±87	2029±20	10280±35	4126±8	9±0.8	2613.5±9.9	301.9±4.8
B188	3356	0.2±0.2	16.2±1.2	14.5±0.8	5684±547	380±10	ND	ND	2.3±0.2	996.9±91.9	23.5±0.9
B189	5504	0.8±0.3	31±0.3	21.3±0.3	17615±1543	571±15	8422±3	1769±36	3±0.3	797.3±53.2	17.4±0.1
B190	8250	6.5±0.1	49.4±3.3	37.4±1.2	23083±871	1395±31	4716±38	1144±53	6.7±0.5	1534.9±111.1	28.5±2.3
B191	17120	55.7±1.3	160.2±0.4	128.5±3.6	46601±717	2153±41	20257±44	14865±401	36.5±5.3	1140.4±52.5	102.4±6.1
B192	12066	87.3±2.1	210±5.9	95±2.3	38622±3331	2637±111	19503±31	9772±219	65±3.8	543.6±25.1	130.3±7.4
B193	20124	63.1±6.3	370.4±8.9	232.9±0.9	64906±5523	3794±63	35447±583	20587±221	69.5±0.4	1840.6±161.4	317±2.9
B194	3203	0.3±0	32.4±0.9	23.3±0.1	7267±62	504±15	381±27	2174±63	7±0.2	151.1±5.3	31.6±1.8
B195	18371	95.9±8	285.9±6.9	146.1±5.8	46486±823	2783±68	26629±667	13624±465	100.3±4	890±37.8	164.9±0.4
B196	22043	120.4±3.6	301.6±27.6	169.4±11.5	54611±551	4538±72	33069±180	21411±384	129±4.1	1782.3±42.1	257.6±15.7

B197	7851	4.6±0.1	28.5±0.7	18.9±0.1	16434±325	161±5	5125±47	238±25	8.4±0.1	873.9±52.9	22.2±2.4
B198	8106	3.2±0.1	60.2±0.1	26.3±0.3	17807±1355	898±27	2584±85	2875±3	14.1±0.3	265.4±19.5	24.5±1.7
B199	3648	20.2±1	51.2±3.2	30.2±0.2	13973±244	533±14	6033±82	2236±83	17.2±1.6	147.4±6.9	66.9±2.9
B200	8260	37.3±0.4	57.7±2.6	41.4±0.4	24040±169	782±19	10884±22	4099±5	8.9±0.4	267.1±17	55.4±2.9
B201	12783	59.6±2.7	122.5±0	85.2±0.5	32760±1014	2528±18	17833±169	12705±199	73.3±4.3	498.4±36.4	69.1±1.4
B202	8304	40.5±2.3	252.1±8.2	68.9±1.7	26016±130	1657±10	12662±334	7499±104	26.7±1.1	419.5±44.5	63.6±1.9
B203	4118	58.5±43.3	29.4±2.7	38.9±1.9	12442±143	707±0	6663±22	2999±49	15±1.5	350.6±33	73.5±5.1
B204	4541	19.6±0.1	40.8±0.1	34.2±1.3	12456±573	686±2	7137±69	2868±57	27±2.1	211.2±10.3	41.3±2.5
B205	11705	69.3±1.8	256±7	128±3	37508±1869	2971±6	21813±819	12243±72	59.4±3.7	853.4±23.3	187.3±0.8
B206	4206	22±0.2	150.4±3.2	95.4±0.4	15531±30	1409±15	9470±119	6005±102	88.3±3.4	770.1±29	168.2±0.7
B207	10904	52.9±2.4	114.1±7.7	103.5±2.9	26741±123	2264±0	16440±240	11264±273	123.7±10.5	775.6±33.4	146.6±12.3
B208	10350	61±0.4	416.8±2.2	97±1.5	33502±384	2592±100	19291±330	9944±28	18.4±0.9	761±46.8	87.6±3.9
B209	4788	27.1±0.4	72.4±0.8	37±0.4	17157±229	832±33	8195±7	2865±44	7.5±0.3	171±10.4	40.9±1.8
B210	17094	57±1.6	132.3±3.7	104.9±3.2	36378±1323	2315±76	21004±174	13155±46	167.4±6.5	1191.8±87	163.2±9.9
B211	12772	58.7±1.3	93.2±2.1	72.6±0.2	33240±653	2106±28	18407±626	7453±10	35.7±0.7	426.7±9.3	191.5±17.4
B212	10257	56±3.9	168.3±4	169.9±12.4	30639±49	3940±33	16025±43	14233±311	239.8±6.8	2008.9±202.3	185.1±10.7
B213	7289	27±0	117.4±0.4	57.4±0.2	23314±20	1341±8	11075±155	5439±112	62.9±5.3	485.4±21.3	111.7±4.2
B214	12821	58.7±2.4	696.7±15.1	101.7±0.2	41133±657	2984±81	21368±22	10490±287	31.4±0.3	462.2±11.7	122.2±13.2
B215	16779	64±3	108.6±3.1	133.8±0.1	32472±1054	2214±22	19086±397	13275±177	147.7±2.8	1038.2±22.9	231.7±1.6
B216	14404	32.4±1.6	155.5±8.1	180.7±7.2	36241±491	3056±24	15860±11	13173±239	9.1±0	931.5±45.3	164.9±5.7
B217	16753	74.8±3.1	151.7±5.2	95.9±0.6	50157±20	2206±33	25663±464	11696±254	63.3±2.6	512±38.7	83.3±8.4
B218	18173	94.2±6.4	164.9±12.7	88.6±2.1	46983±446	2834±24	26045±457	11079±57	40.1±0.3	501.8±11.8	131.8±10.5
B219	8034	22±0.4	60.6±2.6	34.3±1.7	21524±783	877±3	10725±236	4050±60	17.5±0.5	332.6±0.4	110.9±3
N1	9882	55.7±2.9	109.1±3	74.3±0.4	30689±1604	2177±42	18107±290	8620±62	25.8±2.5	544.4±2.8	45.9±0.3
N2	2050	17.8±0	38.9±1.7	39.4±0.4	6627±172	792±15	3191±36	2783±25	12.1±0.2	280.3±1.1	30.3±1.5
N3	1870	28.7±0.3	35.4±0.5	39.6±2.5	13728±150	1159±1	4804±91	4289±97	33.1±0.1	386±2.9	46.5±1.9
N4	7609	26.7±1.6	322.9±2.2	48.4±0.9	17669±1013	871±7	8499±179	6048±33	45.6±0.9	268.1±11	75±3.9
N5	9054	44.8±2.6	89.3±1.3	62.7±1.2	28385±1460	1087±29	10204±163	8036±118	276.4±21.4	502.4±18.2	77.6±3.3
N6	8792	36.7±2.6	637.8±2	80.2±1.4	22837±817	1583±59	11648±111	7018±62	81.1±1.8	658.6±19.9	162.6±9.4
N7	17922	100±4	170.4±0.4	195±7.1	56332±3469	4832±27	33327±160	18676±177	50.8±3.7	1285.5±41.6	192.8±3.9
N8	11599	59.3±5.7	115.5±0.2	82.9±4.8	35567±1280	2332±17	17502±261	8816±311	93.8±1.3	1055.7±46.6	105.3±0.3
N9	3753	11.3±0.2	37±0.5	15.2±0.2	9604±311	480±1	5484±33	1594±8	6.1±0.3	110.8±1.5	45.9±2.9
N10	4750	24.7±1	60.7±2.7	30.4±0.1	15364±249	773±3	7558±37	2841±1	12.6±0.6	182.2±7.1	54.7±3.5

N11	18022	56.2±2.6	131.8±3.8	99.4±2.1	38807±1974	2606±9	20597±248	12076±57	183.9±5.8	1305.3±12.7	146.3±10.4
N12	8557	25.9±0.5	76.9±0.8	53±0.1	17230±1528	932±3	5289±26	5422±77	196.8±2.8	672.3±6.6	138.2±5.2
N13	12357	61±1.3	214.8±3	150.4±4.5	27977±1007	2693±15	14471±230	11912±222	75.8±5.8	934.8±92.6	73.3±6.1
N14	14499	52.9±2.7	164.6±4.1	102.5±9.4	33711±1015	2350±0	22299±190	10912±292	107.6±0.6	597.5±37.2	117.4±1
N15	12335	50.8±2.3	242.5±0.2	73.2±1.1	30715±523	2052±42	14903±15	8596±58	84.9±3.5	628.9±23.1	124.3±7.1
N16	6580	25±1.1	94.7±0.8	41.2±0.2	16696±1037	371±1	8097±108	3897±85	26±0.7	234.2±4.4	84.9±2.7
N17	11105	34.7±0.1	57.9±4.1	32.8±0.5	29221±229	682±7	15811±61	5521±22	17.1±0.9	341.5±27.3	54.8±3.9
N18	13168	62.5±0.5	546.5±7	112.5±5	36868±186	1565±16	17788±271	9358±79	57.4±3.9	678.4±8.6	140.6±9.4
N19	7128	31.7±2.3	157.5±7.1	55.1±1.6	17934±57	1150±12	9527±163	3845±25	23.7±0	250.7±6.1	114.7±9.4
N20	8619	39.4±1.7	65.7±0.1	76.9±1.2	21957±1829	1802±40	10919±12	6149±168	20±1.6	279±25.4	50.9±0.9
N21	19586	74.3±3.4	610.4±9.4	193.4±9.9	55270±696	4998±66	28432±220	18002±97	31.2±2	1250.8±37.2	167±12.3
N22	4744	20.5±0.7	113.7±11.6	34.5±0.2	11915±215	404±0	7013±7	1818±68	5±0.5	126.1±4.8	27±2
N23	6469	23.3±0.1	52.3±0.6	46.9±1.3	15550±270	860±10	10295±11	3465±31	9.9±0.7	213.9±4.6	96.7±2
N24	3328	12.4±0.2	58.1±0.9	19.5±1.1	8769±589	312±8	3601±44	1351±13	9.9±1.1	130.4±5.2	27.6±2.6
N25	10140	43.6±0.7	68.2±5.1	61.6±3.6	26251±2068	1658±7	13994±305	9310±99	57.7±2	544.7±16.8	82.3±5.5
N26	6176	29±1.9	336.1±4.7	52±0.6	18117±303	1334±13	10303±204	4019±22	20.8±1.8	250.9±28.5	42.8±3.4
N27	5170	18.1±1.1	96.6±5.6	40.8±0.1	13203±690	906±8	5856±84	3588±19	18.2±1.1	377.2±51.3	84±0.8
N28	8262	0.7±0	3654.6±47.8	10.6±0.5	2781±47	467±5	8449±66	3900±21	4±0.1	510.7±35.6	47.6±2.2
N29	9944	10.1±0.7	34±1.4	49.7±2.7	21059±225	1953±4	ND	8250±93	26.4±1.8	485.4±17.7	27.2±1.9
N30	12095	76.5±0.3	338.6±2	184.9±2.3	26330±618	3943±9	18027±40	18669±231	112.8±3.7	1378.1±66.3	113.6±3.2
N31	5609	17.9±0.8	158.4±5.3	28.7±0.4	16647±45	761±0	8162±199	3329±10	60.9±4.8	389.7±28.2	82.6±2.1
N32	8548	48.8±4	454.6±26.6	103.8±3.2	23565±419	2171±25	11523±105	9282±58	130.2±5.1	955.3±0.7	115.7±3.4
N33	6515	29.9±0.4	308.8±4.3	72.9±0.8	16885±535	1204±35	7347±107	4698±101	28.8±0.4	479.1±30.9	107.9±1.4
N34	3173	11.4±0.9	205.3±2.5	33.1±0.9	10404±103	472±6	5951±13	2351±44	14.8±1	209.5±6	94.4±4.8
N35	12738	62.9±4.5	223.9±10.1	115.7±1.3	33391±225	2725±20	17577±287	10460±124	35.9±2.9	575.5±36.8	104.2±7.9
N36	10115	28.8±0.2	1599.3±16.7	48±2	17773±169	1151±7	10296±89	3546±43	20.2±0	167.5±13.6	68.6±0.4
N37	1876	15.8±0.9	33.7±2	16.8±0.1	7936±339	565±3	3831±104	2060±11	16.7±0.7	114.3±5	22.4±0.5
N38	10382	45.9±1.2	259.7±0.6	64.9±2.3	29753±1210	1625±15	13405±128	6370±126	15.9±0	555.1±54	70.5±0.1
N39	4764	16.6±0.5	1163.2±7.7	46.9±0.4	12313±672	1139±21	7514±124	4505±138	19.3±0.7	445.4±9.2	70.2±0.7
N40	9124	50.1±2.5	135.7±5.8	94.9±6.9	25668±732	2093±23	13601±358	8213±33	26.5±1.5	510.6±35.2	82.3±2
N41	17880	82.3±0.7	161.6±2.3	133.3±3	40025±404	2542±28	25082±193	12486±114	22.2±0.3	960.1±14.1	123.5±6.1
N42	11380	69.2±1.6	1778.6±58.8	142.5±2.3	33481±205	2630±20	18044±22	9875±128	27.1±0.1	763.5±16.1	136.8±1.2
N43	2250	9.8±0.6	53.1±0.7	20±0.2	7349±132	615±7	1251±46	1799±12	3.1±0.1	117±6.6	49.4±1.8

N44	15584	60.9±1.4	173.9±6.3	129.7±8.5	40355±1566	1970±24	23638±403	10871±107	182±8.9	897±18.6	115.4±7
N45	8654	26.2±1.7	190.7±1.1	45.7±1.2	22379±745	1292±2	12058±224	4502±72	9.2±0.7	374.5±30.1	31.3±1
N46	5518	23.9±1.1	258±3.2	44.8±2.5	19670±319	1121±8	11448±53	5183±17	42.9±2.8	366.4±19.7	87.2±3.7
N47	8157	29.5±2.3	66.6±0.6	31.8±0.3	18950±552	1082±15	11780±314	3309±27	34.6±1.7	198.7±16.4	61.9±4.1
N48	4016	20.9±0.1	68.7±0.8	33±0.9	11762±6	784±9	5735±31	3134±60	19.2±0.1	255.1±7.7	48.1±0.9
N49	10999	100.9±4.5	181.5±2.1	230.6±2.4	31124±1974	5139±45	16816±7	16658±280	27.4±2.3	1741.1±0.4	73.6±1.7
N50	12235	47.2±4.6	110.4±2.2	54.1±0.6	29822±713	1555±14	4879±63	6386±64	13±0.7	327.7±20.6	63.8±3.3
N51	8819	40.9±3.6	79±7.4	62.8±0.4	32877±3138	1694±13	16250±522	8173±57	66.5±2.3	719.9±35	99.2±9.3
N52	1437	7.9±0.2	274±0.1	20±0.3	4954±318	408±4	1129±7	1245±14	30.4±1.9	197.8±3.6	54.7±2.4
N53	8993	108.6±4.7	217.6±14.4	345.5±7.2	25731±619	8503±11	16209±243	20523±309	37.8±0.3	3301.1±92.5	78.1±1.1
N54	9371	33.6±2.1	188.5±1.7	59.9±0.6	25237±66	1242±4	13097±36	5705±4	48.1±2.8	478.1±7.9	82.3±7.7
N55	21165	136.5±4	209±6.9	189.9±6.8	55729±194	4747±137	27632±375	19787±228	22.1±0.2	1143±93.2	193.3±9
N56	8098	40.5±3.1	124.8±6.8	56.6±0.3	22950±256	1386±34	11131±287	5192±1	36.8±2.3	315.1±12.4	55.2±0.8
N57	10993	61.4±3	277.6±5.4	116.6±0.2	34320±1565	2364±58	18367±62	9816±59	22.2±1.4	721.5±14.4	82.7±3.7
N58	24190	126.3±0	319.2±22.8	243.3±0.5	55600±809	6001±3	33083±988	22618±356	51.8±1.9	1508.9±141.4	111.4±1.1
N59	3077	15.1±0.4	38.9±1.2	28.9±0.7	10456±163	658±12	5623±60	2419±8	22.7±0.1	189.5±8.6	44.2±0
N60	7489	31.7±2.8	124.5±6.8	37.8±1.8	22065±41	1317±23	9741±45	5693±26	43.4±2.8	455.9±8.5	59.2±5.1
N61	14132	38.7±1.7	228.8±1.9	50.4±1.1	41094±555	2008±25	22292±3	7770±133	20.4±0.4	435.9±33	117.7±10.4
N62	12479	65.7±2.4	118.5±1.2	97.1±1.7	36429±1011	1914±19	20407±462	9749±309	251.8±9.8	1066.1±23.2	147.5±4.5
N63	24494	22.7±1.7	408.8±19.7	99.4±0.3	58314±528	3917±25	32131±87	15324±44	19.6±0.1	1178.5±91	137.6±11.8
N64	17942	126.1±7.3	436.5±2.3	378.3±34.7	43031±494	7950±197	27191±719	23362±518	15±0.4	2010.8±117.4	189.2±15.2
N65	26519	120±6.2	255.8±2.7	345.9±20.1	64680±924	5818±20	36244±1253	22138±484	12.2±1	1528.9±85.1	105.4±9.6
N66	32960	126.5±8.3	224.9±0.5	253.8±8	82117±3437	6838±74	40513±7	29754±502	82.5±7.4	1265.7±39	147.9±7.3
N67	4803	17.7±1.1	109.9±0.8	24.8±0.3	14977±70	795±13	7810±316	3172±13	53.4±1.7	283.9±24.6	59.6±5.9
N68	18257	69.8±0.8	152.3±1.3	79.7±0.1	35991±98	2173±6	22420±598	10094±150	187.3±8.8	1119.7±79.6	178.9±4.7
N69	7899	49.4±0.5	60.5±5.8	65±0	23074±608	2108±58	12238±69	7457±25	25.6±1	469.6±30.1	45.5±1.5
N70	11396	20.9±1.2	89.8±5.3	22.6±0.4	22542±1140	1058±35	11636±40	4111±42	46.5±4.3	342.4±33.9	67.9±6.6
N71	16834	69.5±1.3	132.3±1	120.6±5.1	42806±1288	2437±12	23520±6	10377±353	94.3±4.3	705.4±41.2	100.3±1
N72	19551	94.9±2.1	137.5±1.1	65±0.2	61626±540	3464±8	33805±189	12930±68	21.2±1.7	684.3±42.2	39±1.7
N73	8948	52.3±3.4	96.6±2.7	92.1±1.8	27638±733	1927±23	16817±182	7556±141	25.9±1.6	402.4±0.4	66.6±0.4
N74	10254	52.1±1	126.7±12.6	109.6±0.3	29179±616	1620±29	16494±442	7836±40	15.9±1.3	480.6±3.2	50.8±1.5
N75	9281	34.1±1.7	98.3±0.4	55.4±0.2	20462±59	1632±31	12243±3	6442±100	27.2±1.2	459.4±36.1	136.7±10.2
N76	7603	82.9±2.1	211.1±2.4	115±8.5	31704±962	3107±38	15765±637	10881±40	35.1±0.1	1011±32.6	47.7±2

N77	10247	61.5±4.6	225.5±4.6	103.5±2.2	35077±749	2490±40	14748±389	12669±118	22.7±1.1	1072.4±59.4	21.8±0.5
N78	20315	97.9±7.8	164.6±3.8	94.7±2.8	54288±1768	3478±30	32322±294	14571±331	54.5±1.2	704.6±55.6	70.5±4.6
N79	3792	7.5±0	12.8±0.9	28.3±0	15316±119	804±1	ND	2970±56	14.7±1.4	214.9±6.7	24.4±1.8
N80	5961	34.1±0.6	77.3±2.1	41.6±0.8	17811±1668	1181±11	9259±105	4539±45	36±0.5	291.2±18.2	63.2±3.8
N81	6063	25.9±2.3	195.8±3.2	42.1±0.1	13839±975	1188±12	7249±48	4578±12	52.2±3	574.5±17.5	68±4
N82	2120	14.2±0.3	40.9±1.9	22.1±0.2	5539±336	520±19	2617±39	1839±17	11.6±0.4	178.4±0.8	21.9±1.1
N83	18092	102.9±6.8	170.6±1.6	110.3±0.4	50061±1834	3374±15	27744±111	12613±30	25±0.7	661.8±30.8	123.6±2
N84	9336	55.2±0.8	148.9±1	105.5±1.3	31238±701	2353±39	13938±171	9127±227	22.2±1.1	525.4±17.9	141±13.3
N85	3841	20.9±0.1	150.4±3.2	35.9±1.1	10982±222	804±27	6136±35	2960±111	14.9±0.8	378.5±13.5	59.3±0.1
N86	7965	47.2±0	50.2±2.5	75.3±2.2	23229±941	1521±15	10797±225	5264±35	39.3±3.3	488.2±43.2	70.1±1.8
N87	15908	97±1.8	277.7±15.7	103.4±1.9	51933±966	3488±97	29570±81	12312±55	63±2.8	794.1±11.7	157.1±11.9
N88	18184	42.1±3	437.3±3.9	78.1±5	39694±147	2283±16	19444±463	7830±103	21.7±0.1	579.2±42.7	68.4±4.3
N89	10157	49.6±1.4	258.6±2.9	56.3±0.2	26788±210	1765±62	15959±346	6809±49	87.6±6.7	549.6±3	87.5±6.1
N90	19859	210±11.2	119.3±2.3	259.8±0.7	62956±1291	6140±105	33558±215	19982±77	15.1±0.2	1384.5±5.1	180.5±7.6
N91	17938	108.6±9.7	123.4±7	102.7±0.6	53352±1609	3520±5	27690±74	14762±7	33.4±1.6	628.7±45	83.5±3.6
N92	17182	108.5±1.2	143.6±5	98.6±0.1	52424±5040	4217±31	28654±830	13072±39	20.7±1.1	511.8±10.6	85.8±6.7
N93	3945	26.2±1.1	48.9±0.4	31.3±0.3	11886±471	1008±31	6436±10	3287±53	19.7±1.9	182±12.4	72.6±7.2
N94	7035	34.8±1.3	310.5±2	71.7±4.2	17448±478	1285±10	8927±72	5430±88	84.3±1.6	646.4±17.6	61.1±2.4
N95	5965	40.2±0.8	123.6±1.7	50.3±0.5	17757±635	1266±7	9862±137	3928±10	8.2±0.4	162.6±12.4	41.2±0.4
N96	7854	40.1±1.7	839.9±15.2	45±0.5	26193±42	1516±2	14505±27	5943±21	73.2±1.4	414.6±30.9	97.1±2.8
N97	9826	38.1±0.5	163.4±1.8	126.2±1.8	30280±813	2137±6	12041±144	11116±411	126±6.1	1167.5±20.7	111.7±0.5
N98	9254	37.9±1	224.5±0.3	47.2±0.7	25781±974	1516±22	12106±313	5778±7	24.4±1.5	407.6±1.2	43.6±3.8
N99	9238	25.8±0.9	74±5.3	43.4±0.2	23107±1008	1497±35	12206±201	3929±44	16.5±0.6	220.6±14.9	72±0.8
N100	6161	35.5±3.1	63.7±1.4	51.6±0.9	18289±144	1128±25	11982±260	5150±85	30.4±1.9	266.8±17.8	37.5±2.9
N101	7679	119.4±5.4	146.1±4.6	158.3±2.3	57232±2774	4055±43	36295±129	14457±431	42.9±1.7	673.8±64.3	109±2.4
N102	11954	17.6±0.4	54.1±3.4	32.1±1.5	11547±410	711±9	6121±175	2548±21	17.1±1.1	175.1±16.2	52.4±0.9
N103	5015	38.8±1.8	392±8.1	47.3±0	25728±65	1607±19	13444±102	5964±42	20±0.4	292.8±20.9	70.9±4.6
N104	11777	5.7±0.1	237.3±3.8	26±0.3	3660±311	499±2	1603±29	1421±14	3±0.2	300.2±24.9	41.9±0.4
N105	7437	38.6±1.1	99±1.1	60±0.9	14630±1	1015±10	7809±18	4858±19	101.3±0.9	461±23.8	67.4±2.8
N106	4392	51.5±0	107.6±0.7	65.6±0.7	29755±856	1776±42	13420±127	6150±118	16.7±1.4	252.3±5.9	69.8±2.6
N107	6639	1.4±0	159.6±2.8	11±0.1	4622±312	204±6	2401±106	779±29	1.7±0.1	130±4.8	46.7±1.7
N108	19931	45.1±0.2	105.5±9.3	56.9±2.2	21484±736	1389±21	11497±250	5206±66	16.1±1.4	255.1±17.6	58.6±4.3
N109	3203	10.8±0.8	13.4±1.2	48.6±0.3	13138±892	637±2	4815±23	522±2	3.6±0.3	1882.1±29.2	56±1

Table S8. The cutoff values of A_m, m^6A, m^1A, m^6A_m, G_m, m^1G, C_m and m^5C.

	A_m	m^6A	m^6A_m	m^1A	G_m	m^1G	C_m	m^5C
Q1	2.77[a]	7.64	5.08	2132	124	837	536	1.19
Q3	5.10	23.6	9.62	2973	214	1527	864	5.26

[a]nmol/mmol Cr

Q1: the first quartile, Q3: the third quartile

Table S9. Univariate and multivariate analysis of risk factors of early-stage breast cancer.

Factors	Univariate analysis			Multivariate analysis		
	OR	95% CI	*p* value	OR	95% CI	*p* value
Age (years)						
⩽ 50	1			1		
> 50	2.423	1.421-4.130	**0.001**	2.812	1.434-5.513	**0.003**
BMI (kg/m^2)						
⩽ 24	1					
> 24	1.791	1.013-3.165	**0.045**			
Menarche age (years)						
⩽ 12	1					
> 12	0.752	0.406-1.574	**0.005**			
CA153 (U/mL)						
⩽ 25	1					
> 25	0.849	0.118-6.131	0.871			
A_m (nmol/mmol Creatinine)						
< 2.77	1			1		
2.77-5.10	0.258	0.122-0.544	**< 0.001**	0.342	0.130-0.901	**0.030**
> 5.10	0.093	0.039-0.220	**< 0.001**	0.123	0.038-0.401	**0.001**
m^6A (nmol/mmol Creatinine)						
< 7.64	1			1		
7.64-23.6	0.281	0.140-0.566	**< 0.001**	0.348	0.139-0.874	**0.025**
> 23.6	0.255	0.116-0.559	**0.001**	0.257	0.092-0.716	**0.009**
m^1A (nmol/mmol Creatinine)						
< 2132	1					
2132-2973	0.204	0.096-0.430	**< 0.001**			
> 2973	0.157	0.068-0.363	**< 0.001**			
m^6A_m (nmol/mmol Creatinine)						
< 5.08	1					
5.08-9.62	0.539	0.282-1.031	0.062			
> 9.62	0.463	0.220-0.974	**0.042**			

G_m (nmol/mmol Creatinine)						
< 124	1					
124-214	0.405	0.206-0.797	**0.009**			
> 214	0.238	0.109-0.517	**< 0.001**			
m^1G (nmol/mmol Creatinine)						
< 837	1			1		
837-1527	0.222	0.103-0.479	**< 0.001**	0.337	0.127-0.899	**0.030**
> 1527	0.083	0.034-0.200	**< 0.001**	0.250	0.081-0.770	**0.016**
C_m (nmol/mmol Creatinine)						
< 536	1					
536-864	0.459	0.237-0.887	**0.021**			
> 864	0.315	0.148-0.671	**0.003**			
m^5C (nmol/mmol Creatinine)						
< 1.19	1					
1.19-5.26	0.306	0.152-0.616	**0.001**			
> 5.26	0.213	0.096-0.472	**< 0.001**			
